# *In Silico* Prediction Analysis of Idiotope-Driven T–B Cell Collaboration in Multiple Sclerosis

**DOI:** 10.3389/fimmu.2017.01255

**Published:** 2017-10-02

**Authors:** Rune A. Høglund, Andreas Lossius, Jorunn N. Johansen, Jane Homan, Jūratė Šaltytė Benth, Harlan Robins, Bjarne Bogen, Robert D. Bremel, Trygve Holmøy

**Affiliations:** ^1^Department of Neurology, Akershus University Hospital, Lørenskog, Norway; ^2^Institute of Clinical Medicine, University of Oslo, Oslo, Norway; ^3^Faculty of Medicine, Department of Immunology and Transfusion Medicine, University of Oslo and Oslo University Hospital Rikshospitalet, Oslo, Norway; ^4^EigenBio LLC, Madison, WI, United States; ^5^Health Services Research Unit, Akershus University Hospital, Lørenskog, Norway; ^6^Adaptive Biotechnologies, Seattle, WA, United States; ^7^Centre for Immune Regulation, University of Oslo, Oslo, Norway

**Keywords:** multiple sclerosis, idiotope, B cell, T cell, bioinformatics, immunoglobulin heavy variable, immunosequencing, immunoglobulin

## Abstract

Memory B cells acting as antigen-presenting cells are believed to be important in multiple sclerosis (MS), but the antigen they present remains unknown. We hypothesized that B cells may activate CD4^+^ T cells in the central nervous system of MS patients by presenting idiotopes from their own immunoglobulin variable regions on human leukocyte antigen (HLA) class II molecules. Here, we use bioinformatics prediction analysis of B cell immunoglobulin variable regions from 11 MS patients and 6 controls with other inflammatory neurological disorders (OINDs), to assess whether the prerequisites for such idiotope-driven T–B cell collaboration are present. Our findings indicate that idiotopes from the complementarity determining region (CDR) 3 of MS patients on average have high predicted affinities for disease associated HLA-DRB1*15:01 molecules and are predicted to be endosomally processed by cathepsin S and L in positions that allows such HLA binding to occur. Additionally, complementarity determining region 3 sequences from cerebrospinal fluid (CSF) B cells from MS patients contain on average more rare T cell-exposed motifs that could potentially escape tolerance and stimulate CD4^+^ T cells than CSF B cells from OIND patients. Many of these features were associated with preferential use of the IGHV4 gene family by CSF B cells from MS patients. This is the first study to combine high-throughput sequencing of patient immune repertoires with large-scale prediction analysis and provides key indicators for future *in vitro* and *in vivo* analyses.

## Introduction

Multiple sclerosis (MS) is a chronic inflammatory, demyelinating, and neurodegenerative disease of the central nervous system (CNS), thought to be mainly mediated by the immune system (1). Although T cells as mediators of disease have been investigated thoroughly over the years, recent trials of B cell targeted therapies (i.e., rituximab and ocrelizumab) point to these cells as equally important contributors (2, 3). Notably, depleting B cells in the periphery has a substantial effect within the CNS (4). It is also possible that other approved therapies for MS act by depleting or prohibiting CD19^+^, CD27^+^ memory B cells from invading the CNS (5). In MS, B cell immunoglobulin heavy chain variable (IGHV) repertoires suggest that clonally expanded plasma cells in the brain and cerebrospinal fluid (CSF) are derived from peripheral B cells that have matured in cervical lymph nodes (6–8). Hence, it seems that peripheral memory B cells play an important role in MS immunopathology.

**Figure 1:**
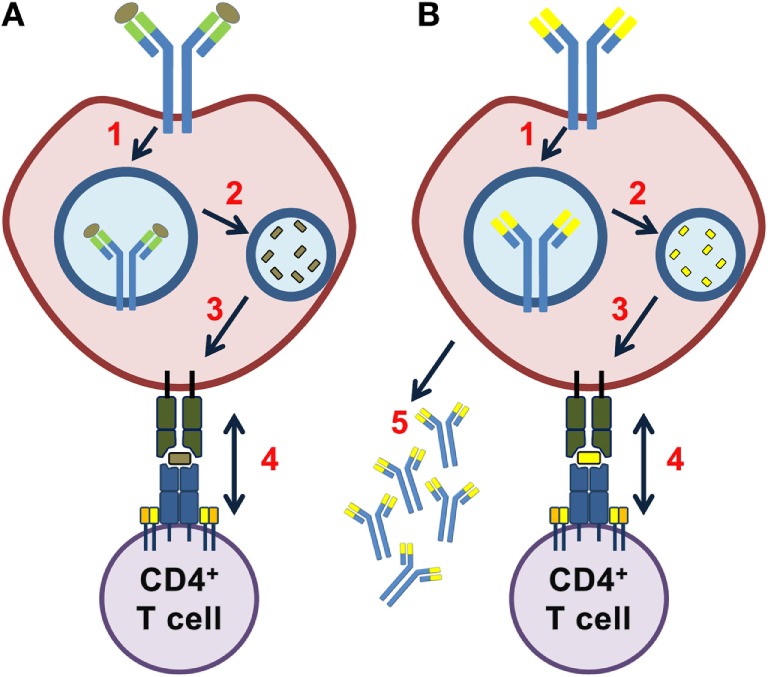
Idiotope driven T–B cell collaboration. In a classical T–B cell collaboration **(A)**, an exogenous antigen bound to the B cell receptor (BCR) is brought into the endosomal pathway (1), processed by proteases (2), and fragments of the antigen presented on human leukocyte antigen (HLA) class II molecules (3). A CD4^+^ T cell specifically recognizing this exogenous antigen provides help to the B cell (4). **(B)** In idiotope driven T–B cell collaboration, a BCR of any specificity (including self) is brought into the endosomal pathway (1), the BCR processed by endosomal proteases (2) and fragments from the variable region presented on HLA class II molecules (3). An idiotope-specific CD4^+^ T cell may help the B cell in a non-linked mechanism (4). All of steps 1–4 must occur for idiotope-driven T–B cell collaboration to take place and may result in differentiation of B cells into immunoglobulin G (IgG) secreting cells (5).

The mechanisms by which memory B cells induce pathology could involve antibody production or secretion of cytokines (9). However, B cell-depleting therapies targeting CD20 ameliorate disease before reducing immunoglobulin G (IgG) production (10), which is therefore not likely their main mechanism. Whereas the discovery of intrathecal Ig production in the CNS is an old one (11), a common antigenic determinant has yet to be discovered. However, B cells in MS lesions (12) and CSF (13–17) have evidently undergone somatic hypermutation indicating T cell help, suggestive of a possible antigen being involved with B cells as antigen-presenting cells (APC) (18). We have proposed an alternative hypothesis to explain how T–B cell collaboration in absence of a common antigen can result in intrathecal IgG production (19). It was shown that B cells present endogenously processed variable region fragments (idiotopes) on major histocompatibility complex (MHC) class II molecules (20, 21). T cells can specifically recognize this idiotope–MHC complex, resulting in a T cell response (22, 23). Such an interaction between idiotope^+^ B cells that present idiotope-MHCII and idiotope-specific CD4^+^ T cells is named idiotope-driven T–B collaboration (21, 24, 25) (Figure 1). An important feature of idiotope-driven T–B collaboration is that unlike conventional antigen-linked T–B collaboration (26, 27), it is unlinked in the sense that while the B cell can recognize any (self) antigen, the T cell recognizes a different antigen (idiotope-MHCII). Thus, B cells of theoretically any specificity, including self-specificity, can be helped by idiotope-specific CD4^+^ T cells to develop into IgG producing plasma cells (25). Consistent with this idea, endogenous idiotopes were eluted from MHC II molecules on B cells (28, 29), and idiotope-driven T–B cell collaboration has been shown to drive the development of autoimmune disease in transgenic mice (30).

Extending idiotope driven T–B collaboration to humans, we have previously demonstrated that human leukocyte antigen (HLA)-DR restricted CD4^+^ T cells from blood and CSF of MS patients can recognize multiple idiotopes within the complementarity determining region 3 (CDR3) and mutated framework (FW) regions on autologous CSF IgG (31–33), showing that MS patients have a repertoire idiotope-matched T–B cell pairs. Idiotope-specific CD4^+^ T cells specifically recognized idiotopes presented by autologous Epstein Barr virus transformed CSF B cells, suggesting that B cells can process and present their endogenous idiotopes on HLA class II molecules (33), and they are also induced to kill oligodendrocytes upon activation (34).

Further large-scale investigations into this mechanism in MS have been hampered by overwhelming numbers of possible IGHV region idiotopes. High-throughput sequencing now offers a possibility to characterize the immune repertoire in unprecedented depth and detail (35) and has triggered a rapid growth of bioinformatic approaches for diagnostic and research purposes (36), including methods to assess possible immunogenicity of B cell variable region sequences (37).

There are several prerequisites for idiotope-driven T–B cell collaboration: the idiotopes would need to undergo endosomal processing; the processed idiotope fragments must have sufficient affinity for HLA class II molecules; and they must be sufficiently rare to avoid T cell tolerance (Figure 1). In this article, we combined high-throughput sequencing of the B cell receptor (BCR) transcriptome with *in silico* prediction analysis to assess whether these prerequisites exist in the intrathecal compartment of MS patients.

## Materials and Methods

### Patients

In this study, we included 11 relapsing-remitting MS patients and six patients with other inflammatory neurological disorders (OINDs), recruited at Akershus University Hospital and Oslo University Hospital. All patients (except MS-11) and the procedures for PBMC isolation, RNA extraction and cDNA preparation have been described previously (7). The cDNA sequencing was performed by Adaptive Biotechnologies using the immunoSEQ level assay (38), with resulting 130bp sequences spanning the IGH-VDJ region.

Multiple sclerosis patients were either treatment naive or treated with first-line therapies (MS-2, MS-3, and MS-4), while OIND patients were untreated at the time of lumbar puncture. All MS patients and one OIND patient had oligoclonal IgG bands in CSF. A summary of patient and sample characteristics are shown in S1 in Supplementary Material.

All participants provided written informed consent for participation. The study was approved by the Committee for Research Ethics in the South-Eastern Norwegian Healthy Authority (REK Sør-Øst S-04143a), the Norwegian Social Science Data Services (no. 11069) and the review boards at AHUS and OUS.

Genotyping for HLA-A, HLA-B, HLA-DRB1, HLA-DQA1, HLA-DQB1, HLA-DPA1, and HLA-DPB1 was performed with four-digit resolution at the Department of Immunology and Transfusion Medicine at Oslo University Hospital, by utilizing a combination of sequence-specific primer- and sequencing based typing technologies. For some patients (MS-1, 3, 5, 8, OIND-1, 5, and 6), we used the strong linkage disequilibrium with HLA-DPB1 to deduce their likely DPA1 alleles (39). HLA types are shown in Table 1.

**Table 1:** Patients HLA types.

	HLA class I alleles		HLA class II alleles				
Patient	HLA-A	HLA-B	DRB1	DQA1	DQB1	DPA1	DPB1
MS-1	02:01 + 02:01	15:01 + 44:02	13:02 + 15:01	01:02 + 01:02	06:02 + 06:04	01:03/05 + 01:03/05^a^	04:01 + 04:01
MS-2	02:01 + 02:01	07:02 + 27:05	01:01 + 15:01	01:01 + 01:02	05:01 + 06:02	01:03 + 01:03	04:01 + 04:02
MS-3	03:01 + 25:01	07:02 + 18:01	15:01 + 15:01	01:02 + 01:02	06:02 + 06:02	01:03/05 + 01:03/05^a^	04:01 + 04:01
MS-4	03:01 + 24:02	07:02 + 35:01	01:01 + 15:01	01:01 + 01:02	05:01 + 06:02	01:03 + 01:03	02:01 + 20:01
MS-5	02:01 + 30:01	13:02 + 44:03	07:01 + 15:01	01:02 + 02:01	02:02 + 06:02	01:03/05 + 01:03/05^a^	04:01 + 04:01
MS-6	03:01 + 32:01	07:02 + 08:01	03:01 + 13:01	01:03 + 05:01	02:01 + 06:03	01:03 + 02:01	01:01 + 04:01
MS-7	24:02 + 69:01	35:01 + 44:02	11:04 + 15:01	01:02 + 05:05	03:01 + 06:02	01:03 + 01:03	04:01 + 04:02
MS-8	01:01 + 31:01	07:02 + 15:01	15:01 + 15:01	01:02 + 01:02	06:02 + 06:02	01:03 + 01:03^a^	02:01 + 02:01
MS-9	25:01 + 31:01	18:01 + 44:03	07:01 + 08:01	02:01 + 04:01	02:02 + 04:02	01:03 + 01:03	02:01 + 04:01
MS-10	03:01 + 29:02	35:01 + 45:01	01:01 + 04:05	01:01 + 03:03	03:02 + 05:01	01:03 + 01:03	04:01 + 04:02
MS-11	02:01 + 02:01	15:01 + 40:01	04:04 + 11:01	03:01 + 05:05	03:01 + 03:02	01:03 + 02:02	02:01 + 05:01
OIND-1	01:01 + 68:01	08:01 + 35:03	03:01 + 15:01	01:02 + 05:01	02:01 + 06:02	02:01/02/03:02 + 01:03^a^	01:01 + 03:01
OIND-2	01:01 + 02:05	08:01 + 50:01	03:01 + 07:01	02:01 + 05:01	02:01 + 02:02	01:03 + 01:03	04:02 + 104:01
OIND-3	01:01 + 01:01	08:01 + 08:01	03:01 + 03:01	05:01 + 05:01	02:01 + 02:01	02:01 + 02:01	01:01 + 14:01
OIND-4	02:01 + 02:01	15:01 + 27:05	01:01 + 04:01	01:01 + 03:01	03:02 + 05:01	01:03 + 01:03	04:01 + 04:02
OIND-5	03:01 + 68:01	07:02 + 44:02	07:01 + 15:01	01:02 + 02:01	02:02 + 06:02	01:03/03:01/04:01 + 02:01^a^	04:02 + 11:01
OIND-6	01:01 + 01:01	08:01 + 08:01	03:01 + 03:01	05:01 + 05:01	02:01 + 02:01	02:01/02/03:02 + 1:03/03:01/04:01^a^	01:01 + 04:02

*^a^Deduced by DPB1 linkage disequilibrium*.

### Preparation of Datasets

After removing non-productive sequences, IGHV amino acid sequences were deduced using the ImMunoGeneTics (IMGT) database and the IMGT/High-V-Quest analysis tool (version 3.3.4) (40, 41). This analysis identified additional non-productive sequences that were removed. IGHV transcripts comprising more than 0.5% of total reads within each compartment were designated “highly transcribed.” Finally a single FASTA file containing all the IGHV sequences with tagged information consisting of patient code, compartment, frequency rank and tag describing whether it was highly transcribed was prepared. These sequences are deposited online at http://doi.org/10.6084/m9.figshare.5035703.

An extensive public dataset of IGHV nucleotide sequences from three healthy individuals (42, 43) was obtained online (http://datadryad.org/resource/doi:10.5061/dryad.35ks2). The corresponding IGHV amino acid sequences were deduced according to IMGT standards, and used for further analysis.

The compiled patient dataset of IGHV amino acid sequences was submitted to EigenBio (WI, USA) for processing and prediction analysis. Each sequence was given a unique general identifier (gi), and sequences with exact matching amino acid sequence within each patient were identified and given a clonal identifier for statistical purposes. Every possible 15-mer and 9-mer (denoted as IGHV fragments) were derived from each IGHV amino acid sequence, and indexed according to their N-terminus CDR3-relative position as determined by IMGT standards (44), designating cysteine 104 at the start of CDR3 as position 0. This indexing process resulted in extensive databases of overlapping IGHV fragments offset by one single amino acid, and provided a basis for systematic comparison of fragments in the FW3 and CDR3 regions across MS and OIND patients, and healthy individuals.

### Cathepsin Cleavage Probabilities

Peptidase cleavage by cathepsins S, L, and B was predicted with neural network models developed using datasets from Biniossek et al. (45), by methods described previously (46). In short, all IGHV sequences were converted into sequential octamers using the P4P3P3P1-P1′ P2′ P3′ P4′ convention with the scissile bond designated as the bond between amino acid 4 and amino acid 5 designated P1P1′. The neural network ensembles each produce a probability of cleavage of the scissile bond ranging from 0 (uncleaved) to 1 (cleaved). An ensemble median cleavage probability of >0.8 was set as the prediction threshold for the analyses.

Endosomal enzymes digest proteins into peptides of varying lengths and a peptide 15-mer is commonly presented on HLA class II (47). However, the HLA class II molecules display peptides of widely varying lengths. In an effort to simulate this process, a “fuzzy logic” system was devised where peptide excision probability was determined by examining the simultaneous cleavage probabilities from the N-terminus minus three amino acids to the C-terminus plus three amino acids. Thus, the process generates predicted excised peptides ranging from 15 to 21 amino acids. The cutoffs for this “fuzzy logic” excision prediction were intentionally lowered to avoid under-prediction. If the maximum probability of cathepsin cleavage at either terminal was ≥0.5 and simultaneously had a probability of ≥0.25 on the other end, that would lead to an “*Excision*” call.

### HLA Affinity Predictions

For each CSF-derived 9- and 15-mer peptide, we predicted the affinity for 37 HLA class I and 28 class II molecules using previously described models (37, 48, 49). The neural network ensembles used for affinity predictions were developed using public datasets of allelic affinities [half-maximal inhibitory concentration (IC_50_) units] from http://www.iedb.org (downloaded June 2012). The alleles for which affinities were predicted are shown in S2 in Supplementary Material. Predicted affinities are either presented as the natural logarithm of IC_50_ [ln(IC_50_)] or a Johnson SI standardized value of ln(IC_50_) within patient and compartment. Standardization was performed to bring the predicted values onto an equal scale for comparative and/or illustrative purposes. In previous publications using such predictions, standardizations were performed within protein, as all peptides within a protein may compete with each other in terms of HLA affinity (37, 46). For the current publication, the IGHV sequences were shorter, and there is also a possibility of HLA affinity competition between different IgG molecules, hence the overall within patient standardization.

### T Cell-Exposed Motifs (TCEMs)

In a peptide-HLA (pHLA) complex, some amino acids of the peptide will be exposed to T cells (TCEM), and others will be oriented inwards toward the HLA molecule (groove-exposed motif). For a 15-mer peptide in a pHLA complex, we numbered the amino acid residues from −3 to 12. Utilizing the work of Rudolph et al. (50) and Calis et al. (51), as previously described (37) three different types of non-continuous TCEM were deduced and designated TCEM I (amino acid residues 4, 5, 6, 7, 8 of a 9-mer), TCEM IIa (2, 3, 5, 7, 8 of a 15-mer with a core 9-mer), and TCEM IIb (−1, 3, 5, 7, 8 of a 15-mer with a core 9-mer). The latter two are relevant for HLA class II predictions, the former for HLA class I predictions. Analysis of the models of Rudolph et al. (50) indicate that TCEM IIa or TCEM IIb motifs occur in approximately equal proportions of TCR:MHC class II structures. All possible TCEM patterns were identified for all IGHV fragments.

Rare IGVH sequences may escape tolerance and be stimulatory under the right circumstances (52). TCEM frequencies are unequally distributed in the IGHV region and also elsewhere in the proteome as some motifs occur far more frequently than others (37). Their frequency in the IGHV region can be assessed by assigning a frequency class (FC), which is a reverse log2 scale where FC 0 (1/2^0^) corresponds to “occurring in every IGHV sequence” and FC 21 (1/2^21^) to “occurring once every approx. 2 million sequence” (37). In this study, we considered a TCEM with FC above 16 (once every 65,536 sequence) as rare.

To compare the mean FC of our patients TCEMs, we compiled a FC classification system using a public database of 37 million unique BCRs spanning the FW3 and CDR3 from memory and naive B cells from three healthy donors published by Dewitt et al. (42, 43), consistent with a previously published classification based on 56,000 sequences from Genbank (37). The IGVH sequences of this database are of the same length and were established with similar technology as those from our patients, thereby minimizing technical or disease-related bias. All TCEMs occurring in the dataset were assigned a FC class based on their mean patient and compartment-specific −log2 frequencies.

The FC as a measure of presumed likelihood for IGVH sequences to escape tolerance does not take into account the possible occurrence of similar TCEMs elsewhere in the human proteome or in the gut microbiome. While the human proteome is common for patients, and can to some degree be accounted for, the gut microbiome displays variations across populations and ages (53). For this publication, we used databases of TCEM occurrences in the human proteome (assembled from UniProt, with removal of Ig variable regions) (54) and microbiome (from NIH Human Microbiome Project Reference Genomes database) (55) as described previously (56), by searching for all 3.2 million possible variations of each TCEM.

Each TCEM occurs at a characteristic frequency in proteomes. These frequencies were then normalized to a zero mean unit variance scale using Johnson SI scale transformation of log2 frequency values.

### Validation of Prediction Analyzes

We have previously derived two monoclonal antibodies from CSF B cells of two MS patients (CSF mAbs), and demonstrated that one idiotope from each of these mAbs (pMS1 and pMS2) was both processed, presented on HLA class II molecules and recognized by cloned CD4^+^ T cells *in vitro* (32, 33). These were therefore suitable for validation of the prediction analyzes. Cathepsin cleavage, HLA affinity, and TCEM occurrence were predicted as for the main dataset. The FC was calculated using a previously described dataset comprising the complete IGVH region (37, 56). Idiotope pMS1-VH1 was presented on DRB1*13:02 and pMS2-VH3 on DRB1*13:01 encoded HLA molecules. As these have identical amino acid sequences, the affinity for DRB1*13:02 was predicted for both peptides.

### Statistics

All predictive models were built by EigenBio using JMP^®^ software version 12.1/13.0 (SAS Institute, Cary, NC, USA), by script processing. STATA v 14.1 (StataCorp LLC, TX, USA) and JMP^®^ 12.1 were used for statistical analyses. Plots were created in JMP^®^ 12.1. All plots displaying CDR3 relative positions are cropped to include ~99% of the IGHV fragments.

For bioinformatics processing purposes, to avoid end-effects in various algorithms, we added a standard immunoglobulin signal peptide and three amino acid sequence (“DTR”) to the beginning, and a 26 amino acid sequence derived from the IgG1 constant region (“GTLVTVSSASTKGPSVFPLAPSSKST”) at the end of each IGHV sequence. Parts of these were retained as described below for the comparative statistical analyses. Our IGHV fragments were indexed by the position of the first cysteine determining the start of the CDR3 region, and changes due to mutations in the CDR3 region could influence both TCEM and affinity predictions at indexed positions even prior to position 0.

For statistical testing, we created three subsets of the IGHV sequences from our patients. For comparison of differences in mean FC and mean HLA affinities between MS and OIND patients within the CDR3, we used a subset limited to fragments with approximately half of the amino acids of the IGHV-fragment within the CDR3. This subset contained fragments starting at indexed position −7, and ended in the position where the fragment would contain eight amino acids of the added constant region. A second subset containing FW3 (spanning approximately amino acid 73–104 by IMGT numbering) and CDR3 regions were compared within MS and OIND patients. For this purpose, we used the patients dataset, with the addition of the “DTR” amino acid sequence at the start and “GTL” amino acid sequence at the end, and defined fragments as being influenced by CDR3 changes similarly as above, with a cutoff at position −7. In the third subset, we used a similar approach as for the second subset, and included the blood-derived sequences for comparisons between blood and CSF. No extra amino acid sequences were attached to the sequences of this dataset.

In the first subset, the differences between patients and controls, between low and high FC within patients and controls, and between IGHV4 and the other IGHV families were assessed by estimating the multilevel mixed effects model for each outcome variable. A multilevel approach was chosen because the data exhibit a three-level hierarchical structure, with levels for patient, relative CDR3 position, and clone. The intraclass correlation coefficient was calculated to assess the cluster effect on each level. The cluster effect was highest at patient-level for all variables. Clone-level demonstrated negligible or no cluster effect and was therefore not taken into account. Adjustment for cluster effect on CDR3 relative position-level caused convergence problems. Therefore, the differences between the categories were assessed by estimating a linear mixed model with fixed effects for factor variable and random intercepts for patients at each relative CDR3 position separately. Benjamini-Hochberg adjustment for multiple testing was applied within each outcome variable with acceptable false discovery rate (FDR) set to 20% (57).

In the second subset, the cluster effect on protein clone-level was also negligible or zero, and hence ignored. The cluster effect on patient- or relative position-level or both were present for most variables. A linear mixed model with fixed effect for factor defining the region was estimated. Random effects for either patients or relative position were included in the model. For variables with cluster effect on both levels, random effects for relative position nested within the patient were assessed, but as these were negligible only the models with single random effect were estimated.

No cluster effect on protein clone-level was found in the third subset. Intrapatient correlations were close to zero or not present. Consequently, comparisons between blood and CSF within patients and controls as well as within IGHV families for patients and controls separately were performed by independent samples *t*-test at each relative CDR3 position, and *p*-values were further adjusted for multiple testing by Benjamini-Hochberg procedure. As this was an exploratory study also aiming to guide further studies on the proposed mechanism, the FDR was set at 20%.

Finally, generalized linear models with random effects for patients were estimated to assess the differences in number of IGHV sequences containing fragments meeting a set of idiotope criteria between MS and OIND groups, as well as between highly transcribed IGHV sequences and other sequences.

The results are reported as mean differences between the groups with the corresponding 95% confidence intervals (CIs) and *p*-values.

## Results

### IGHV Transcripts

From all patients a total of 1,812,920 IGHV amino acid sequences were deduced after removing non-productive transcripts, with a mean of 3,552 (95% CI 1,399–5,706) sequences obtained from CSF and 125,180 (95% CI 78,262–172,100) from blood. The mean CDR3 lengths of CSF sequences were 15.55 amino acids (95% 15.5–15.6, *n* = 25,556) for MS patients and 15.25 amino acids (95% CI 15.20–15.29, *n* = 34,833) for controls (*p* < 0.001, independent samples *t*-test). For blood, the corresponding lengths were 15.48 (95% CI 15.48–15.49, *n* = 1,551,154) for MS and 16.03 (95% CI 16.01–16.04, *n* = 201,377) for controls (*p* < 0.001, independent samples *t*-test). The IGHV gene family usage is shown in Table S3 in Supplementary Material. As reported previously by us and others (7, 58), there was a preferential use of IGHV4 in CSF from MS patients.

Most IGHV sequences and transcripts used in this project were previously published (7). However, in the present study we also included exceedingly rare sequences for the purpose of creating a thorough database of TCEM, resulting in a higher total number than previously described (7).

### Cathepsin Cleavage

**Figure 2:**
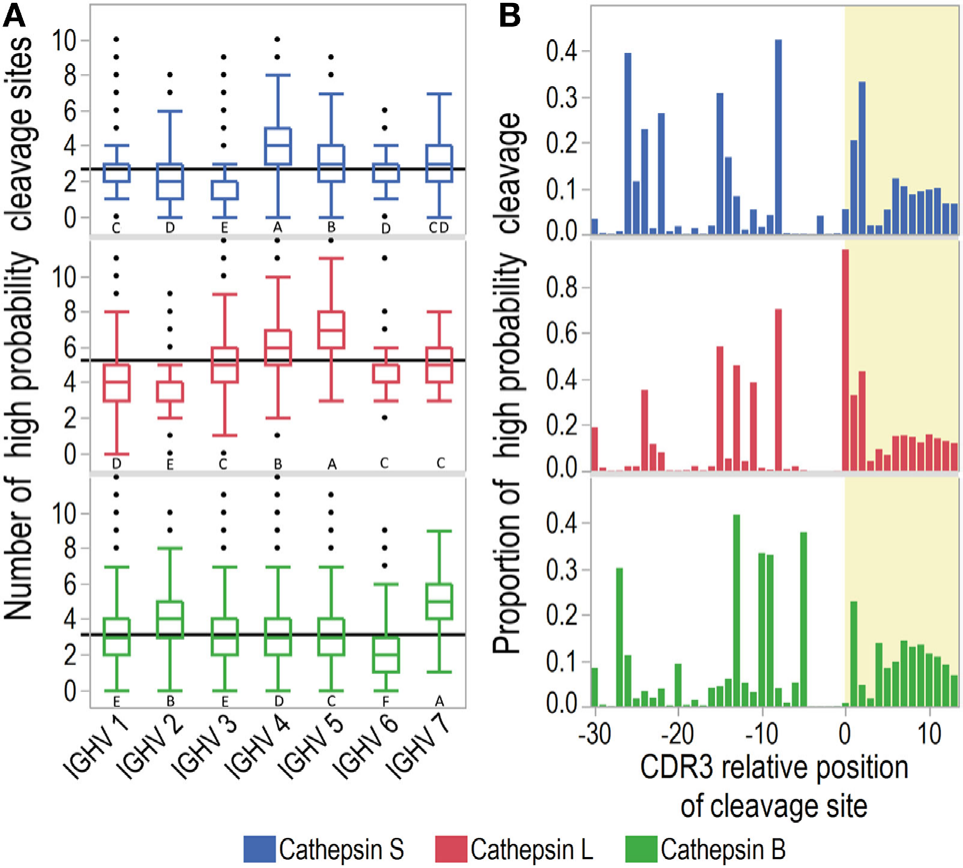
Prediction of cathepsin cleavage. Cathepsin cleavage sites in immunoglobulin heavy chain variable (IGHV) transcripts were predicted with neural-net models. **(A)** Mean summarized numbers of predicted cleavage sites (>0.8 probability for cleavage) for transcripts of all IGHV families are shown as solid black lines and distributions as outlier box plots with whiskers covering first and third quartile ±1.5*(interquartile range). For each cathepsin, IGVH families not connected by the same letter are significantly different (Tukey Kramer HSD). **(B)** The proportion of transcripts with >0.8 probability of cleavage for each complementarity determining region 3 (CDR3) relative position. The CDR3 relative position aligned with the cleavage site at P1-P1′. CDR3 is marked with yellow shading.

Due to the IGHV4 bias in CSF among the MS patients, we first investigated whether the predicted pattern of cathepsin cleavage differed across IGHV families. An analysis of variance by IGHV family yielded significant variations (Welch ANOVA F(6, 951.32/951.49/949.68) for cathepsins S, L, and B, respectively, *p* < 0.0001 for all). Cathepsin S was predicted to preferentially cleave IGHV4 derived sequences, cathepsin L was predicted preferentially to cleave those from IGHV5, and cathepsin B to preferentially cleave those from IGHV7. Interestingly, cathepsin S was also predicted to cleave IGHV3 sequences least efficiently (Figure 2; Table S4 in Supplementary Material).

As CDR3 is most diverse and therefore hypothesized to be the main source of immunogenic idiotopes, we further investigated whether the cathepsins were likely to release CDR3 fragments (Figure 2B). All three cathepsins displayed a similar overall pattern of cleavage sites in FW3 and at the start of CDR3. Notably, cathepsin L was predicted to cleave almost all IGHV sequences before or after the cysteine marking the start of CDR3. Cathepsins S and B showed less pronounced peaks for cleavage at the same positions.

**Figure 3:**
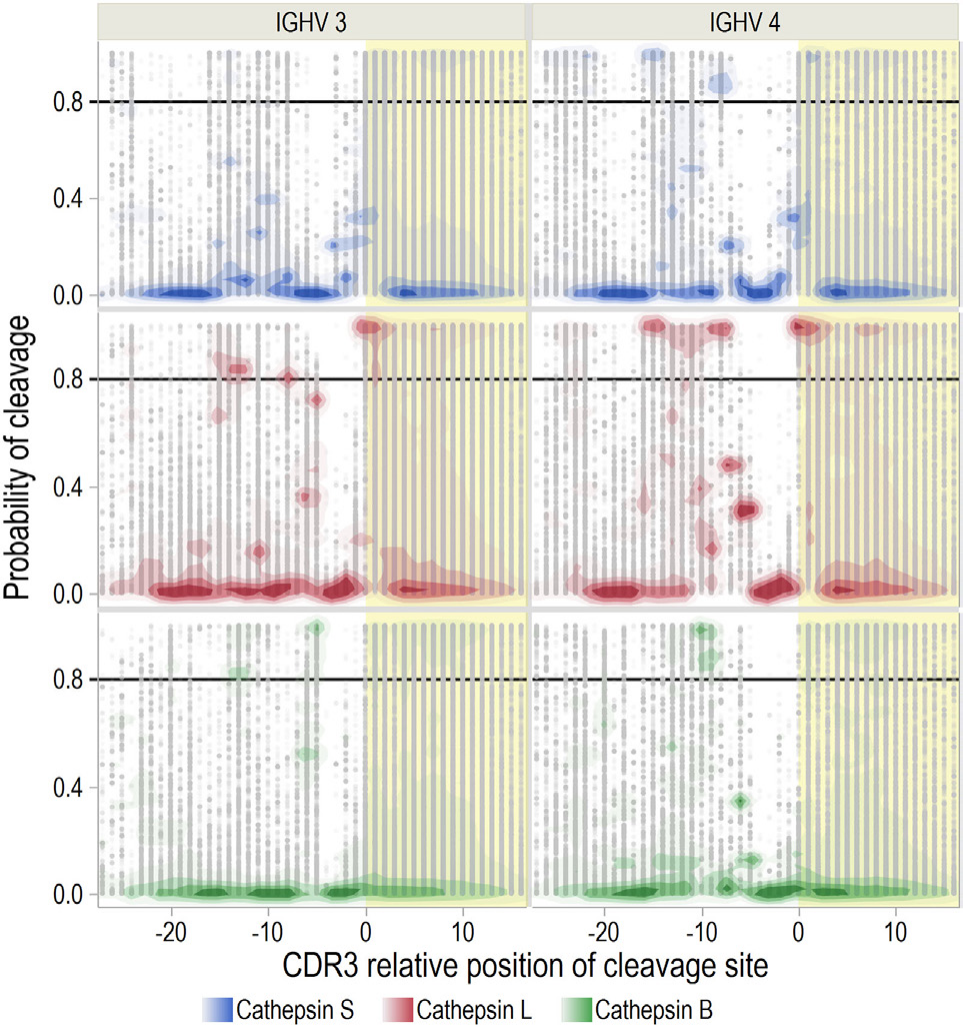
Distribution of cleavage probabilities by complementarity determining region 3 (CDR3) relative position. Cathepsin cleavage sites in immunoglobulin heavy chain variable (IGHV) sequences were predicted with neural-net models. The distributions of predicted probabilities for cleavage (range 0–1) are shown for IGHV3 and 4 (see S5 in Supplementary Material for IGHV1, 2, and 5–7). Sites with predicted probability >0.8 are considered to have high probability for cleavage. The CDR3 region is marked with yellow shading and the CDR3 relative position is aligned with the cleavage site at P1-P1′.

We next investigated whether these patterns differ across IGHV families (Figure 3; S5 in Supplementary Material). The previously identified hotspot for predicted cathepsin L activity at the CDR3 start was consistently found for all IGHV families. For cleavage of IGHV4 by cathepsin L and S, there were also two hotspots in FW3. No hotspot was identified for cathepsin S for IGHV3. As cathepsins S and L are endopeptidases that recognize octamers within the peptide, cleavage at these hotspots would effectively block other cleavages in the immediate vicinity. Therefore, these hotspots probably represent the most likely cleavage sites.

### HLA Affinities

Different HLA molecules may display different binding affinities for IGHV fragments. Analyzing all CSF IGHV fragments together, fragments from CDR3 had consistently higher predicted affinities for HLA-DR and -DP than fragments from FW3 (Figure S6 in Supplementary Material). While DRB1*15:01 was among the DR molecules with the highest mean standardized affinities for CDR3-derived fragments, the same was not true for the linked DQA1*01:02-DQB1*06:02 among DQ molecules. In general, the predicted patterns of affinities for different HLA class II molecules were similar between MS and OIND patients (not shown).

**Figure 4:**
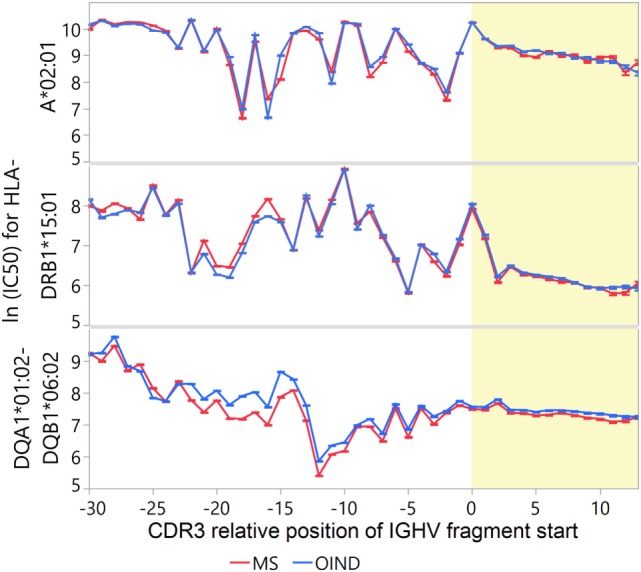
Immunoglobulin heavy chain variable (IGHV) fragment affinities for multiple sclerosis (MS)-associated human leukocyte antigen (HLA) molecules. Binding affinities of cerebrospinal fluid (CSF) IGHV fragments were predicted for HLA-A*02:01, HLA-DRB1*15:01, and HLA-DQA1*01:02-DQB1*06:02 with neural-net models. Mean Johnson SI standardized values of ln(IC_50_) were calculated for each CDR3 relative position, with low values indicating higher affinity. Yellow shade indicates the CDR3 region. Each error bar is constructed using a 95% confidence interval of the mean.

We next investigated the IGVH sequences from CSF for binding affinity to MS-associated HLA molecules (Figure 4). CDR3 fragments from MS patients had higher predicted affinity compared to FW3 fragments for DRB1*15:01 and to a lesser extent for DQA1*01:02-DQB1*06:02, but lower affinity for A*02:01 (*p* < 0.001 for all, S7 in Supplementary Material). After correcting for intraclass correlations at patient-level, there were no significant differences between the MS and OIND patients in predicted affinity for these HLA molecules for any IGHV fragment in vicinity of the CDR3. Similarly, no significant differences were detected for MS or OIND patients when comparing highly transcribed to other IGHV sequences (data not shown). However, IGHV4 family fragments from MS and OIND patients had higher predicted affinity for DRB1*15:01 than other IGHV fragments at almost every position within the CDR3 (Table S8B in Supplementary Material). For DQA1*01:02-DQB1*06:02 and A*02:01, the results were similar to those for DRB1*15:01, except for IGHV4 where both higher and lower mean affinities were predicted within the CDR3 (Tables S8C,D in Supplementary Material).

**Figure 5:**
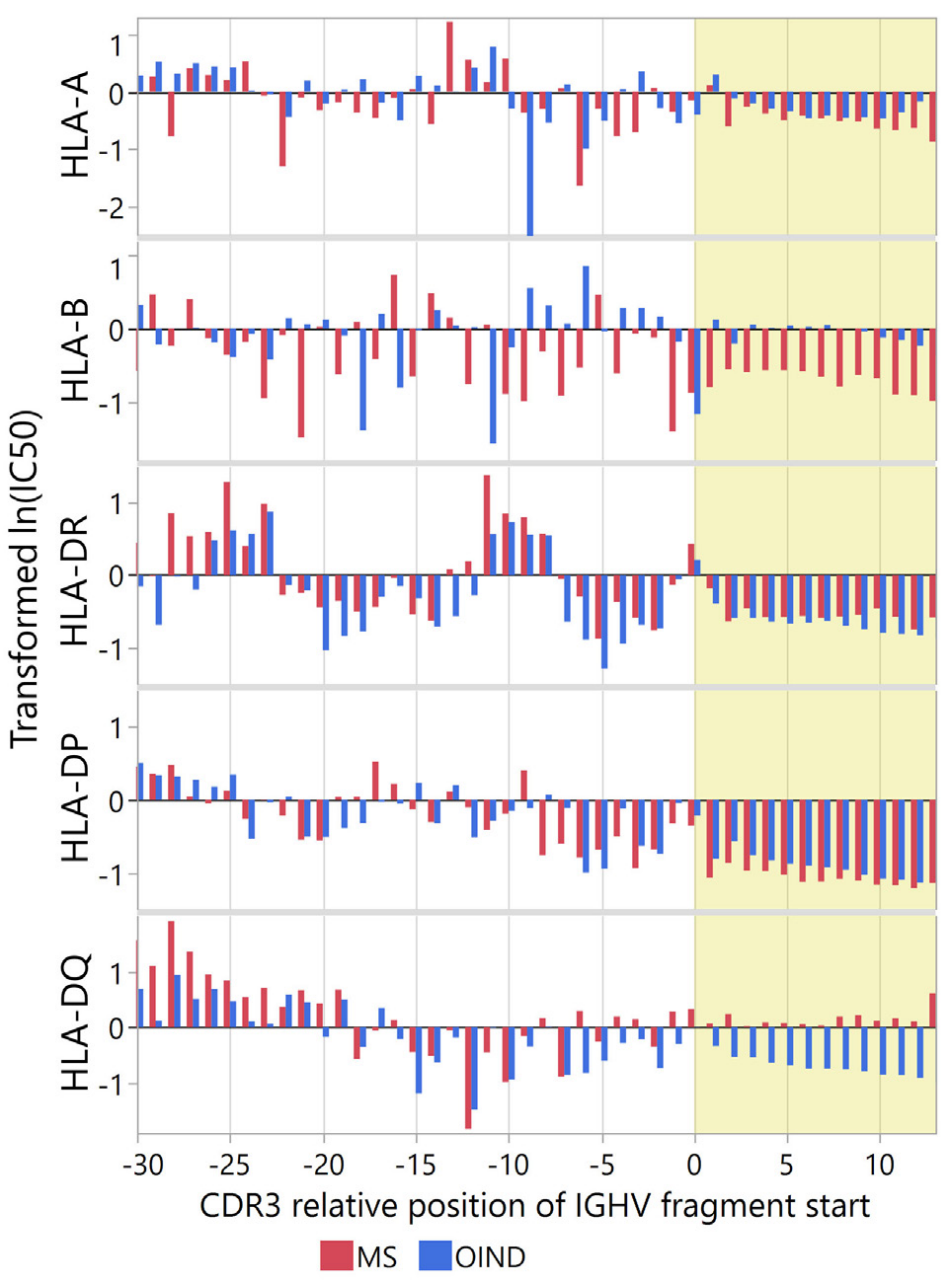
Immunoglobulin heavy chain variable (IGHV) fragment affinities for patient-specific human leukocyte antigen (HLA) molecules. Binding affinities of IGHV fragments were predicted for patient-specific HLA A, B, DP, DR, and DQ molecules (listed in Table 1). Results are presented as mean Johnson SI standardized values of ln(IC_50_) for each complementarity determining region 3 (CDR3) relative position, with low values indicating high affinity. For heterozygous patients with two sets of predictions for one HLA molecule, we used the lowest standardized ln(IC_50_). Yellow shade indicates the CDR3 region.

Because each patient did not carry all HLA alleles, we extracted the affinity predictions for those carried by each individual. For heterozygous patients, we used the allele with the highest predicted affinity (Figure 5). In general, the standardized predicted affinities were very similar for DR and DP, with high predicted affinity in CDR3 for both MS and OIND patients. For DQ on the other hand, only OIND patients followed this pattern. This most likely reflects that MS patients more frequently carry DQA1*01:02-DQB1*06:02, which we previously showed did not display the highest affinities within the CDR3.

### TCEM Patterns

From all possible IGHV fragments (*n* = 52,566,906) we identified TCEM I, IIa and IIb and created databases of TCEM occurrences. We identified approximately 1.5 million unique TCEM of each pattern from a theoretical maximum of 3.2 million. These overlapped >98% with the TCEMs in the dataset derived from healthy individuals by DeWitt et al. (42), which could therefore be used for frequency classification in our dataset (Table 2).

**Table 2:** Number of unique TCEM identified in each dataset.

	Patient dataset^a^	Healthy BCR dataset	Overlap (%)^b^
TCEM I	1,448,203	2,747,840	98.64
TCEM IIa	1,439,194	2,736,223	98.62
TCEM IIb	1,403,685	2,710,199	98.64

*^a^Combination of blood and CSF derived IGHV fragments from both MS and OIND patients*.

*^b^Percentage of TCEM identified in the patients, also identified in the DeWitt et al. healthy BCR set*.

**Figure 6:**
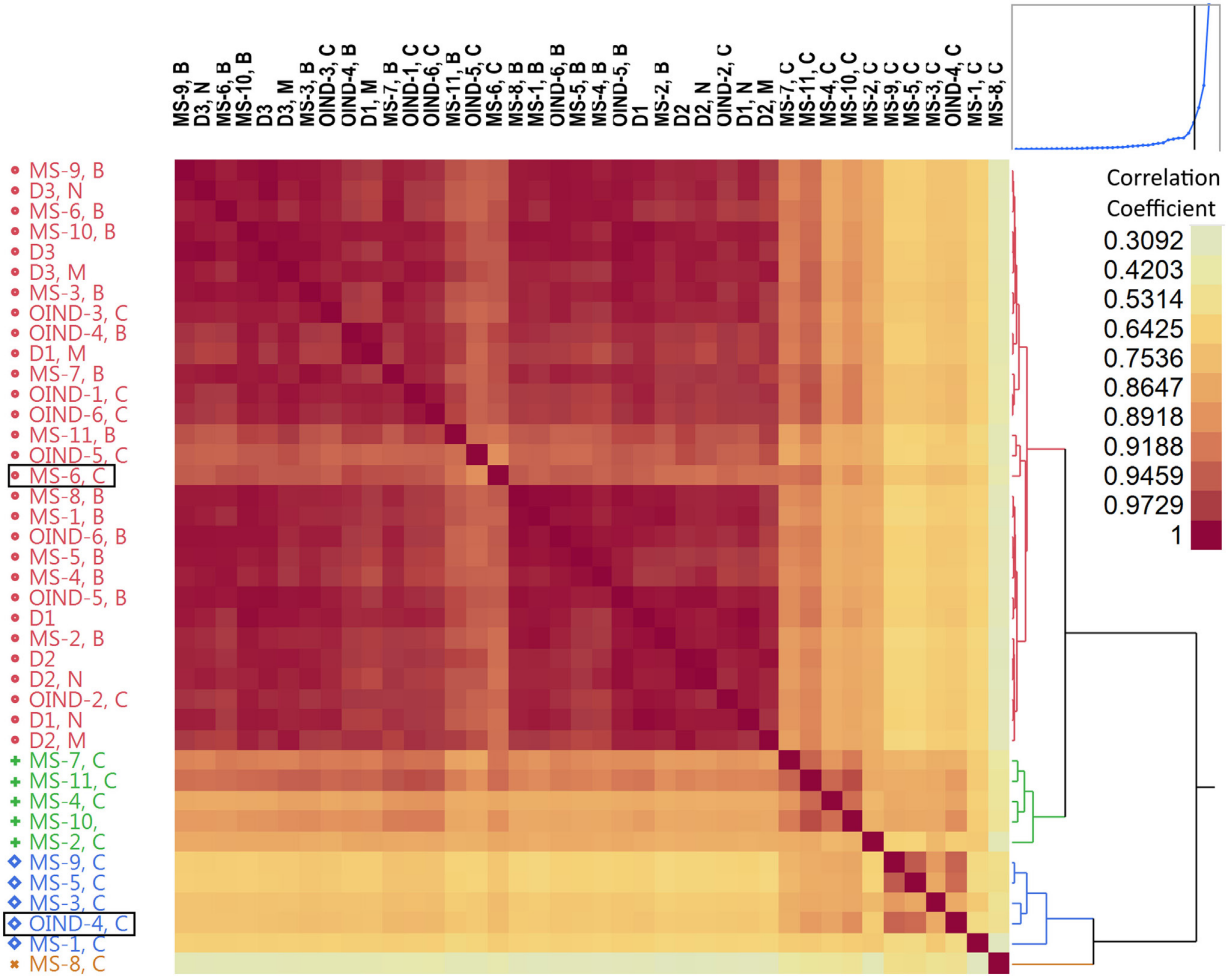
Hierarchical cluster analysis of T cell-exposed motifs (TCEMs) by patient and compartment. The occurrences of all TCEM IIa in immunoglobulin heavy chain variable (IGHV) fragments were identified and summarized by patient and compartment in patients with multiple sclerosis (MS, MS 1–11), other inflammatory neurological disorders (OINDs, OIND 1–6), and in three healthy individuals (D1–3). Pairwise correlation coefficients were calculated for all possible pairs, and displayed as hierarchical cluster matrix. Blood samples from all individuals cluster together with OIND cerebrospinal fluid (CSF) samples (red), while MS CSF generally cluster differently (blue, green and orange). The two exceptions (MS-6 C and OIND-4 C) are marked with boxes. For D1-3, *N* denotes naive B cells from blood, and M denotes mature B cells from blood. For MS and OIND, B at the end of the identification code denotes blood and C denotes CSF.

Different occurrences of TCEM between populations and compartments could point to a selection process. The results of cluster analysis performed on pairwise correlations for the summarized occurrences of TCEM IIa in blood and CSF for each patient group are shown in Figure 6. The TCEM patterns of IGHV sequences from CSF of MS patients differed from those in blood and also from those of the OIND patients. There were two notable exceptions; TCEM from CSF of OIND-4 clustered consistently with that in CSF of MS patients. This patient was the only OIND patient with oligoclonal IgG bands (OCB) and IGHV4 dominance in the CSF. The other exception was MS-6, from whom the TCEM in CSF clustered with that in blood. Notably, MS-6 was one of two MS patients with dominant IGHV3 use in CSF. TCEM I and IIb displayed similar patterns as TCEM IIa (data not shown). Corresponding results were found using standardized (z transformed) TCEM occurrences (S9 in Supplementary Material).

We next performed cluster analysis on pairwise correlations of TCEM occurrences by compartment and IGHV family. This revealed clustering on IGHV family level (S10 in Supplementary Material), implying that either dominance of IGHV4 or relative lack of IGHV3 could be driving the differences in TCEM patterns. This also suggests that differences in number of IGHV sequences were not driving the TCEM clustering, as could be suspected because CSF samples generally clustered together. Similar results were obtained when analyzing samples by IGHV family, patient-ID and compartment. Again the samples clustered mainly by IGHV family and secondly by compartment (data not shown).

### Frequency Classifications

**Figure 7:**
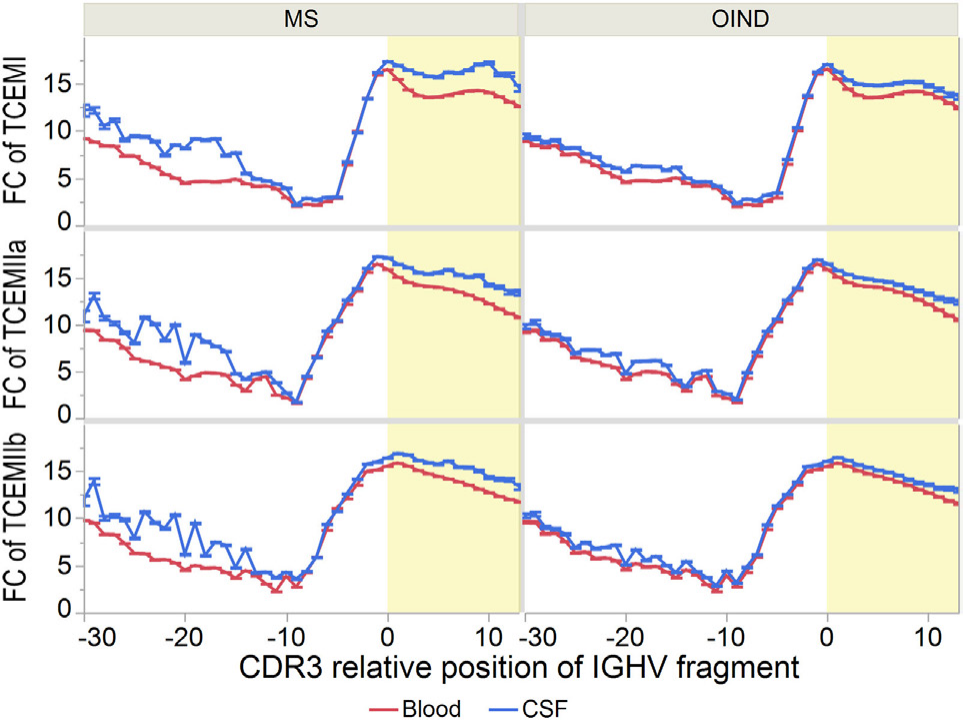
Frequency of T cell-exposed motifs (TCEMs)—differences between blood and cerebrospinal fluid (CSF). Mean frequency class (FC) of TCEMs [−log2 values with 95% confidence interval (CI)] in immunoglobulin heavy chain variable (IGHV) fragments were calculated for all complementarity determining region 3 (CDR3) relative positions and compared between blood and CSF. High FC indicates rare TCEMs. In both multiple sclerosis (MS) and other inflammatory neurological disorders (OINDs) patients, fragments from CSF carry significantly more rare TCEMs than fragments from blood (*p* < 0.001, mixed-model comparisons, see Tables S11A–C in Supplementary Material for details). Yellow shading indicates CDR3.

As CSF IgG is more mutated than IgG from blood (7, 12, 15, 59), the TCEMs of IGHV sequences from CSF could be rarer than those from blood. We therefore compared FC distributions in blood vs. CSF (Figure 7; Tables S11A–F in Supplementary Material). Although most pronounced among the MS patients, the mean FC of TCEMs from the CDR3 was significantly higher in CSF at nearly every CDR3 relative positions for all three TCEM for both patient groups. The mean FC was also significantly higher in CSF for each IGHV family analyzed separately (data not shown).

Our analyzes predicted that CDR3 fragments were both likely to be released by cathepsins and to bind HLA class II molecules with higher affinities than FW3 fragments. Hence, we analyzed whether the CDR3 region generated more rare TCEMs. The CDR3 regions of CSF from both MS and OIND patients generated on average more rare TCEM than FW3 (*p* < 0.001 for all TCEMs), probably driven by greater diversity in the CDR3 (Table S12 in Supplementary Material).

**Figure 8:**
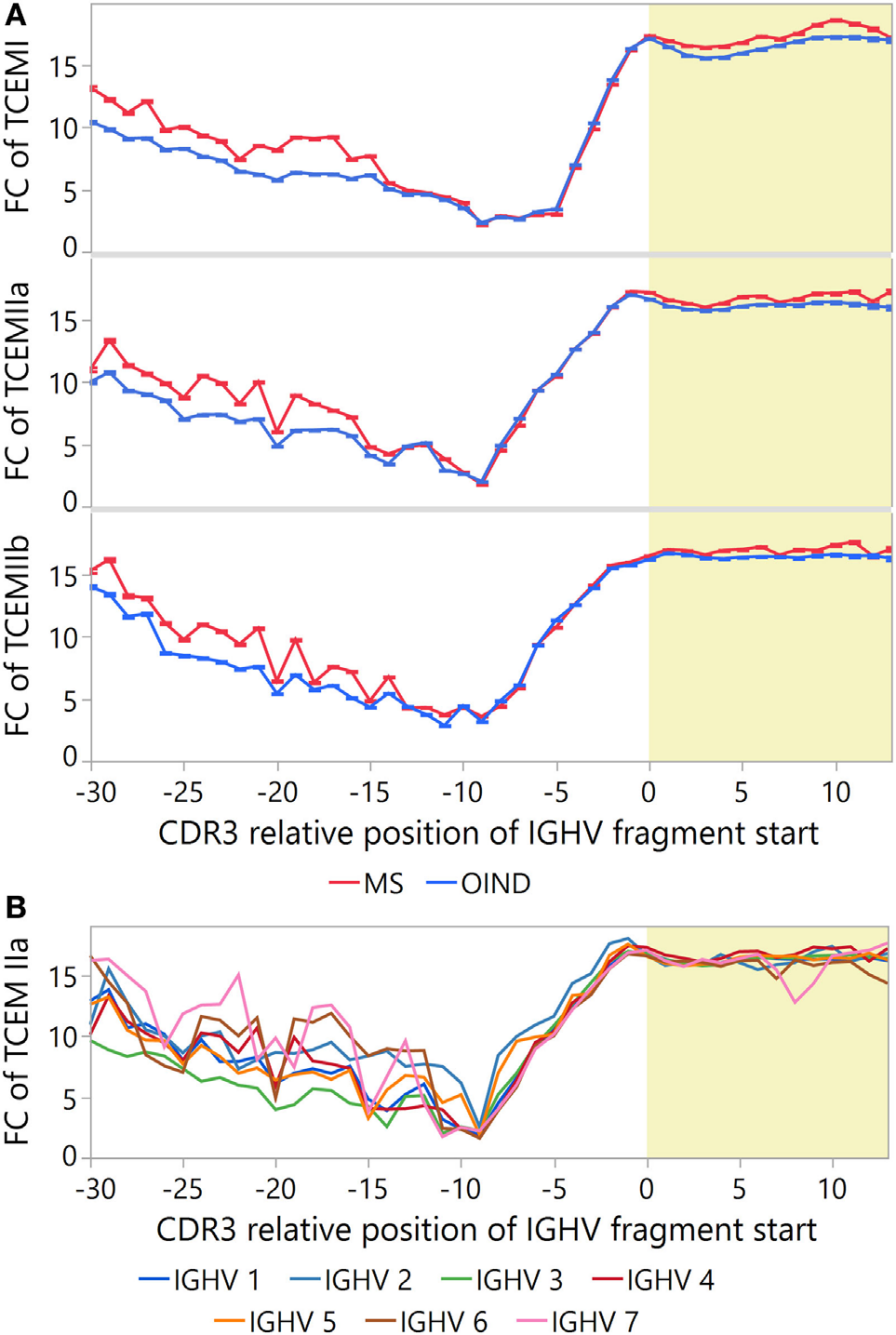
Frequency of T cell-exposed motifs (TCEMs) in immunoglobulin heavy chain variable (IGVH) fragments from cerebrospinal fluid (CSF)—differences between patient groups and IGVH families. Mean frequency class (FC) of TCEMs [−log2 values with 95% confidence interval (CI)] in CSF IGHV fragments were calculated for all CDR3 relative positions, and differences are shown **(A)** between multiple sclerosis (MS) and other inflammatory neurological disorders (OINDs), and **(B)** by the IGHV families. High FC indicates rare TCEMs. Yellow shading indicates CDR3.

Because rare motifs are expected to be most likely to escape tolerance (52), we also tested whether CDR3 fragments from MS patients contain on average rarer motifs than those from OIND patients. After correcting for intra-class correlations in a multilevel hierarchical mixed model and multiple testing, the MS patients had significantly higher FC than the OIND patients at several CDR3 positions (Figure 8A; Tables S13B–D in Supplementary Material). This was most evident for TCEM I and IIa.

As IGHV4 family bias was found in MS CSF samples (Table S3 in Supplementary Material), we compared the FC of sequences carrying IGHV4 to other IGHV families (Figure 8B). Differences in FC between IGHV families were most pronounced prior to position −7. For CDR3, we found statistically higher FC at nearly all positions for sequences carrying IGHV4 than for sequences carrying any other IGHV family (Tables S13E–G in Supplementary Material). Thus, it seems that IGHV4 possess all the assumed prerequisites for T cell stimulation.

### Summarized Attributes

**Figure 9:**
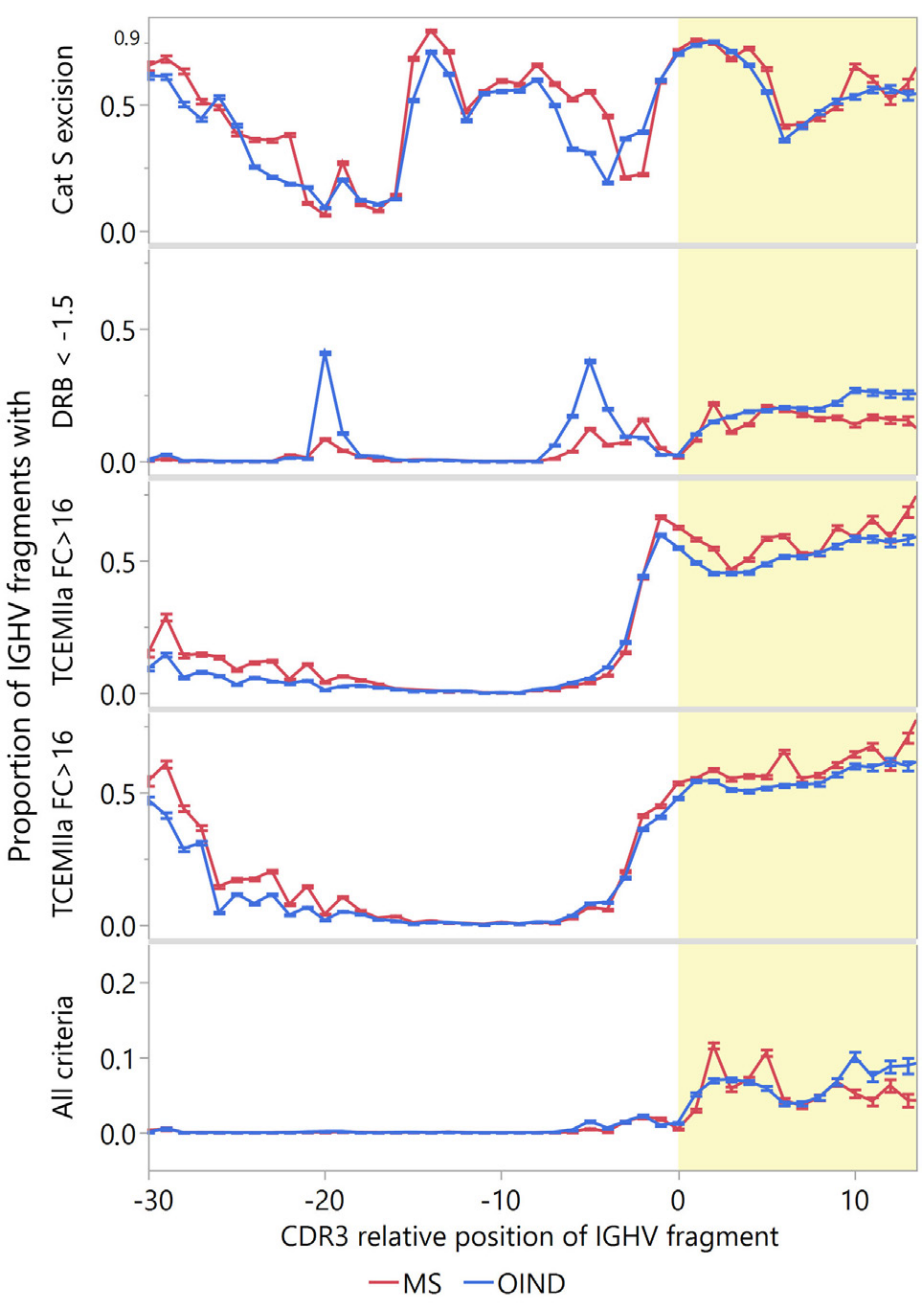
Proportion of immunoglobulin heavy chain variable (IGVH) fragments that could engage in idiotope-driven T–B cell collaboration. The idiotope score identifies IGHV fragments most likely to engage in idiotope-driven T–B cell collaboration in context of human leukocyte antigen (HLA)-DR molecules [excised: predicted 15-mer release by cathepsin S; high affinity: patient-specific Johnson standardized ln(IC_50_) for HLA-DRB1 < −1.5; rare: frequency class (FC) of T cell-exposed motif (TCEM) > 16]. The upper panels show the proportion of fragments at each complementarity determining region 3 (CDR3) relative position that fulfills each criterion. The lower panel shows the proportion that fulfills all criteria. Nearly all fragments inhabiting all three features occur in the CDR3 region (yellow shading). Error bars indicate 95% confidence interval of the mean.

**Figure 10:**
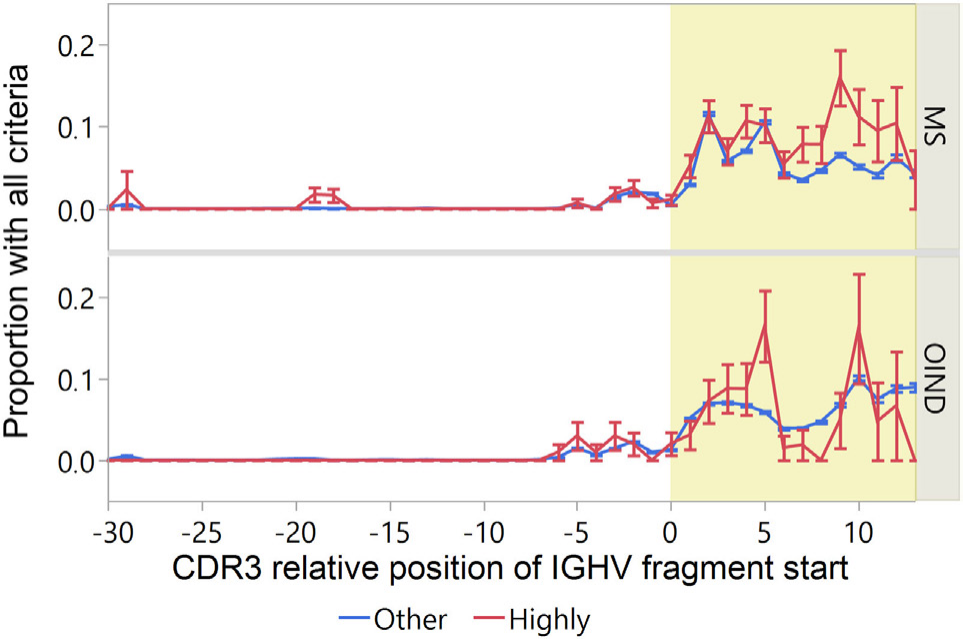
Fragments from highly transcribed immunoglobulin heavy chain variable (IGVH) sequences from multiple sclerosis (MS) patients are most likely to meet the criteria for idiotope-driven T–B cell collaboration. The proportion of IGHV fragments meeting the requirements for idiotope driven T–B collaboration [excised: predicted 15-mer release by cathepsin S; high affinity: standardized human leukocyte antigen (HLA)-DRB affinity < −1.5; rare: frequency class (FC) of T cell-exposed motif (TCEM) > 16] is displayed by their complementarity determining region 3 (CDR3) relative position. CDR3 region yellow shaded. Error bars indicate 1 SEM.

To identify IGVH sequences most likely to engage in idiotope-driven T–B cell collaboration, we devised an “idiotope score” identifying IGHV fragments with both high patient-specific HLA-DRB1 standardized affinity [ln(IC_50_) < −1.5]; TCEM II (a or b) FC > 16; and predicted fuzzy cut “Excision” by cathepsin S (Figure 9). Although many IGHV fragments fulfill one of these criteria relatively few fulfill all, and these almost exclusively reside in the CDR3 region. In a few peaks in the CDR3, almost 10% of the IGHV fragments fulfilled all criteria. Among the MS patients, the highly transcribed CDR3 fragments in CSF were generally most likely to carry all these traits (Figure 10). Moreover, in MS patients highly transcribed IGHV sequences were more likely than others to carry at least one IGHV fragment with these attributes (OR = 1.39, *p* = 0.01 unadjusted model). However, after adjusting for cluster effect on patient-id-level the difference was no longer significant (OR = 1.24, *p* = 0.11). For OIND patients, we found no significant difference in unadjusted (OR = 1.20, *p* = 0.38) or adjusted models (OR 1.25, *p* = 0.29). Among highly transcribed IGHV sequences, 42% from the MS patients and 34% from the OIND patients carried at least one fragment fulfilling all criteria (OR = 1.41, *p* = 0.16; adjusted on patient-id-level: OR = 1.40, *p* = 0.27).

### TCEM in the Human Proteome and Gut Microbiome

By plotting the mean Johnson standardized frequencies of TCEM found in the human proteome and gut microbiome in a similar fashion as the immunoglobulin FC scale, we found that the CDR3 regions generate TCEM that generally occur rarely in both the gut microbiome and the human proteome (S14 and S15 in Supplementary Material). There was a significantly lower standardized mean occurrence of TCEM in the CDR3 than FW3 for both gut microbiome (*p* < 0.001) and the human proteome (*p* < 0.001). Hence it seems that somatic hypermutation and recombination in the CDR3 is capable of generating TCEM that are rare, both in the healthy IGHV repertoire and in the human proteome and gut microbiome.

### Validation

**Figure 11:**
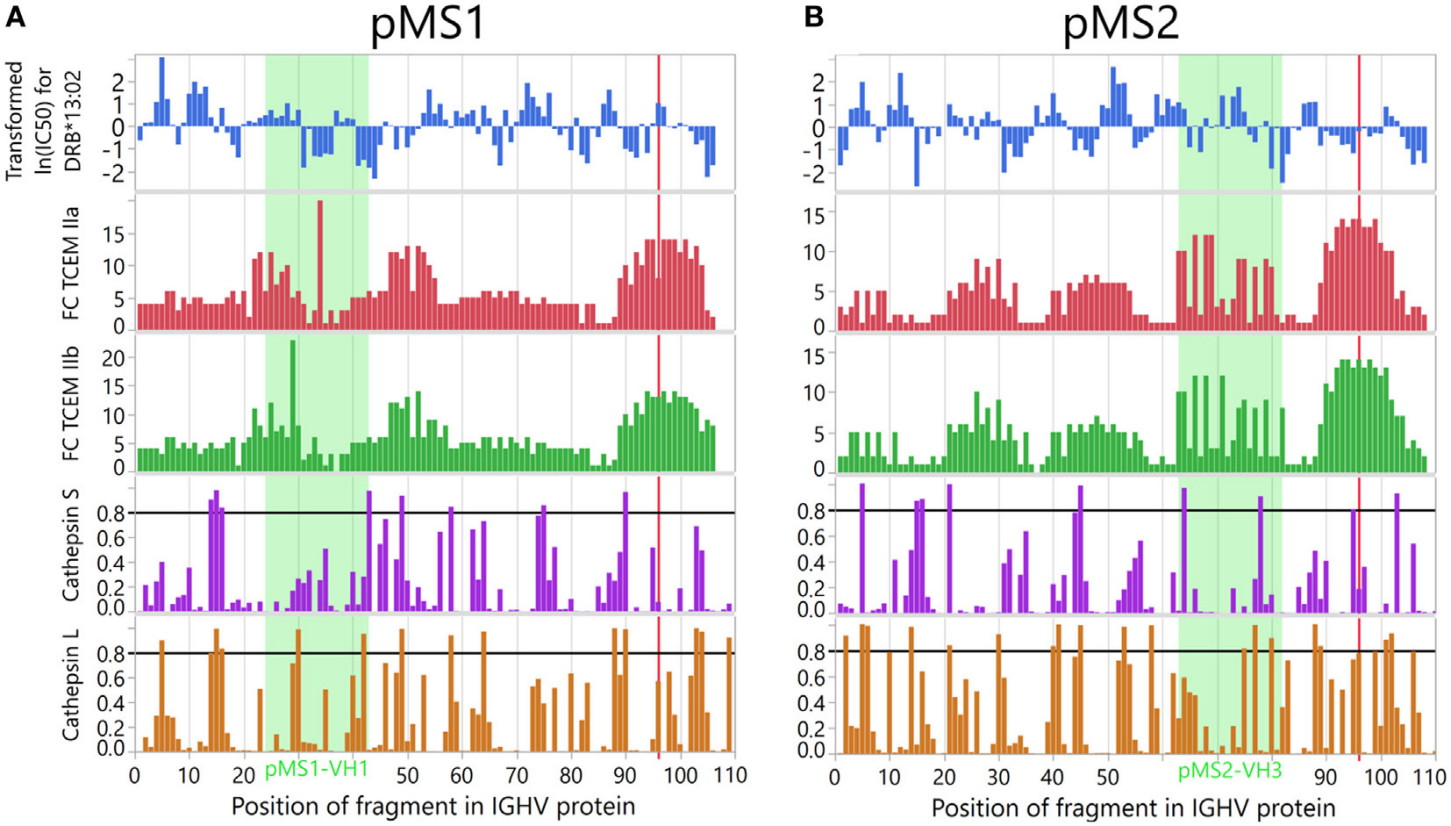
*In vitro* validation of *in silico* prediction analyses. The peptides **(A)** pMS1-VH1 and **(B)** pMS2-VH3 are derived from V regions of immunoglobulin G (IgG) produced by cerebrospinal B cells of two multiple sclerosis (MS) patients and have been shown *in vitro* to be processed and presented on human leukocyte antigen (HLA) DRB1*13:01/13:02 molecules to idiotope-specific CD4^+^ T cells. Cathepsin cleavage, HLA affinity and T cell-exposed motif (TCEM) occurrence were predicted as for the main dataset, for each position within the immunoglobulin heavy chain variable (IGHV) transcript. The frequency classes (FCs) were obtained using a dataset comprising the full length VH region, and are therefore not directly comparable to those calculated for the main dataset. The cysteine at the start of complementarity determining region 3 (CDR3) is marked with a red line.

To validate the epitope prediction, we performed prediction analysis of the V regions of two CSF mAbs previously derived from CSF B cells of two MS patients (32). We have previously demonstrated *in vitro* that each VH region of these CSF mAbs carry an idiotope (pMS1-VH1 and pMS2-VH3) that was processed by APCs, and presented on DRB1*13:01/13:02 molecules to CD4^+^ T cells which specifically recognized the particular idiotope (32, 33). As shown in Figure 11 prediction analysis anticipated that both peptides were likely to be cleaved at positions allowing for presentation by the relevant HLA-DR molecule, and that they would bind their restriction element (DRB1*13:01/13:02) with high affinity. The FCs had to be calculated for the complete VH region and are therefore not directly comparable to those used for the complete dataset, but nevertheless shows that the TCEM associated with these idiotopes are rare.

## Discussion

We have hypothesized that idiotope-driven T–B cell collaboration may drive the intrathecal immune response in MS (19, 33). Although proof of concept studies have provided some evidence compatible with this hypothesis (31–33), the immense diversity of the immune repertoire have previously precluded further analyses. Here we combined high-throughput sequencing of IGHV transcripts with *in silico* prediction analyses to assess whether the requirements for such T–B cell collaboration exist. Our findings indicate that idiotopes from the CDR3 regions of MS patients on average have high affinities for disease associated HLA-DRB1*15:01 molecules and are predicted to be endosomally processed by cathepsin S and L in positions that allows such HLA binding to occur. Additionally, CDR3 sequences from CSF B cells from MS patients contain on average rarer TCEM that could potentially stimulate non-tolerant CD4^+^ T cells, than corresponding sequences from OIND patients. Many of these features were associated with the previously described IGHV4 gene family bias (7, 58, 59) of CSF B cells in MS, indicating a possible explanation for this previously unexplained predominance. The IGHV gene family distribution with IGHV3-gene family dominance in blood also correlated with previously published results from healthy individuals (37, 42, 60).

Cathepsins S and L are essential for antigen presentation on MHC class II molecules (61, 62), including both processing of invariant chain (Ii) and preparation of protein fragments expressed on MHC class II molecules (63). Cathepsin S was shown to be particularly important for endosomal processing in B cells, dendritic cells and macrophages, while cathepsin L was essential for the Ii chain processing in cortical thymic epithelial cells and in macrophages (64, 65). Both these cathepsins are therefore likely relevant for endosomal processing of idiotopes. Cathepsin S was also implicated in MS by early expression studies suggesting a possible disease association (66), but this has not been replicated in more recent GWAS studies (67). The cathepsin S (*CTSS*) gene was further reported to be associated with treatment responses of both glatiramer acetate and IFN-beta (68). It was shown that cathepsin S could cleave myelin basic protein as a possible mechanism of action (69), and a cathepsin S-like helminthic protease was efficient in cleaving IgG (70). Even earlier publications showed that human lysosomal proteases can cleave IgG at acidic and to a lesser extent at neutral pH (71). Cathepsin S and L have similar cleavage patterns in general (72). Our cleavage predictions are in line with these findings and indicate that cathepsin S especially may be important for endosomal processing of BCRs in a way that allows idiotopes of the CDR3 region to be released.

Three observations point to CDR3 as crucial for generation of idiotopes capable of stimulating CD4^+^ T cells: First, cathepsins S and L both displayed high probability of cleavage around the CDR3 start; second, these cleavage spots were immediately followed by regions with high predicted affinity for HLA class II molecules; and third, the same region was associated with relatively high mean FC values for all TCEM, implying high likelihood for T cell stimulation.

It has been shown that IGHV fragments from endogenous IgG are indeed processed and presented on MHC II molecules (20). However, while the effects of cathepsin S and L on foreign antigens and Ii-chain have been well characterized (73), there have been no specific studies to our knowledge on how human cathepsins S and L act on Ig variable regions in endosomal conditions. The predictive models were built on data from proteomic identification of cleavage sites assays (45), where cleavage sites are readily available in preprocessed polypeptide cocktails (45, 46). Native IgG molecules, on the other hand, contain disulfide bonds that could interfere with cathepsin activity (74). Future studies addressing these questions would be important for validating our predictions.

B cells are likely to play a role as APCs in MS (18), but which antigens they present are unclear. B cells are capable of processing and presenting their own Ig, as demonstrated almost 30 years ago in mice (20, 22), and recently shown to occur in large scale on HLA class II molecules in humans with mantle cell lymphoma (75). Such presentation would normally be enhanced by BCR stimulation, which activates B cells and induces proper antigen presentation potential (76). An alternative way for the B cell to upregulate their HLA class II expression and antigen presenting potential, was shown in mice to occur through CD40 stimulation in the thymus (77), as thymus B cells upregulated AIRE, HLA class II and CD80 in a CD40 dependent (and BCR independent) mechanism. It is not known whether B cells from the CNS or CSF of MS patients express AIRE.

Our results of predicted high affinity for HLA class II molecules for CDR3-derived fragments are consistent with findings in the Genbank IGHV dataset from healthy donors, including the different patterns observed for HLA-DQ (37). Notably peptides derived from the CDR3/FW3 region studied here were recently shown to be extensively presented on HLA class II in human mantle cell lymphoma B cells, and also recognized by idiotope-specific CD4^+^ T cells (75). Interestingly, for the single lymphoma patient with available HLA data and T cell specificity (75), our predictions confirmed high affinity of the eluted VH peptide to HLA-DRB1*04:01 molecules [predicted ln(IC_50_) = 3.69, SD = 0.69]. Along with our results, this implies that the diversity of this region either generates on average higher affinity peptides, or that some idiotopes are selected for their affinity to HLA class II. As our sequences did not span the whole Ig variable region, we were unable to compare affinities for peptides derived from CDR3 and FW3 with those derived from CDR1, −2 and other FW regions. However, the recent results from human mantle cell lymphoma suggest that the most relevant part of the Ig molecule for idiotope-driven T–B cell collaboration were included in this study (75).

The concept of idiotope-driven T–B cell collaboration is founded on an idea of lack of tolerance for the IGHV region, but it is not fully known to which extent such tolerance occurs (52). Central T cell tolerance is mediated through positive and negative selection within the thymus, with help of cortical and medullary thymic epithelial cells (mTECs) as well as dendritic cells (78). During negative selection T cells are exposed to self-peptides by mTECs with help of promiscuous gene expression regulated by the autoimmune regulator AIRE protein, leading to either clonal deletion or induction of regulatory T cells (78). However, V (D) J recombinations of the IGHV genes only occur in B cells, and mTECs presumably are unable to present the huge number of idiotopes resulting from this process. Yet it was shown that T cells are likely tolerant to germline-encoded (non-mutated) IGHV regions (23, 79). It is possible that this could be mediated through circulating Ig, as very high concentrations of monoclonal Ig can induce tolerance through clonal deletion in the thymus (80, 81). Recent studies have found that both naive and class-switched B cells in the thymus of mice are of peripheral origin and capable of AIRE-induced antigen presentation (77, 82), even without BCR stimulation. This could provide another explanation as to how B cells can generate tolerance in the thymus, but the studies did not investigate to what extent such B cells present their own IGHV regions. Also, only a few B cells are present in the thymus at any time (77), providing a relatively small pool of BCRs to generate tolerance.

Our study utilizes TCEM as a model for how TCR interact with pHLA. Rudolph et al. described how only a few amino acid residues of the peptide in a pHLA complex interact with TCRs (50). These observations were then used to deduce the atomic contacts of motifs exposed to T cells (37). As HLA class II TCEMs are non-linear, matching TCEM can appear in context of both high and low affinity peptides. The TCEM model is applicable to any protein in a pHLA complex, and we expect TCEM occurring in the human proteome to be associated with tolerance if they are presented in context of HLA (56). A logarithmic scale of TCEM frequency classification (FC system) was developed and described for an IGHV repertoire from healthy individuals, and it was shown that each TCEM has a characteristic frequency of use. Some TCEM occur very frequently in IGHV regions (low FC), while others are incredibly rare (high FC) (37). The observation that some TCEM are present in every single, second, fourth, etc., IGHV sequence could possibly explain why relatively few thymic B cells may induce central tolerance for a substantial proportion of the IGHV repertoire (37). In agreement with previous findings of high diversity in transcribed CDR3 regions from CSF B cells (7), we found here that the CDR3 sequences from CSF contained on average quite rare TCEM, compatible with high likelihood of escaping tolerance. Moreover, the finding that CSF B cells have higher mean FC (more rare) TCEM than blood B cells is compatible with the notion that B cells are selected into the intrathecal compartment for their ability to stimulate idiotope-specific T cells. Finally, our comparison of TCEM in the IGHV sequences and the human proteome and gut microbiome found that CDR3-derived IGHV fragments more frequently carried TCEM that were rare in both these compartments, again suggesting that they would be more likely to escape tolerance. It was previously shown that neither the gut microbiome nor the human proteome cover the entirety of TCEM diversity (56), allowing for occurrence of many TCEM unique to the IGHV regions.

In agreement with the recently discovered lymph drainage of the CNS (83), B cells in the brain of MS patients seem to mature in cervical lymph nodes (8), and B cells in the CSF are clonally related to those in blood (7, 59). B cells may also proliferate within ectopic lymphoid follicles within the CNS of MS patients with long-standing disease (84). Hence, maturation necessary for idiotope-driven T–B cell collaboration could occur both in the periphery and within the CNS (8, 59). Such maturation may increase CDR3 variability and influence any of the parameters investigated in this study, for instance mutations that generate rare TCEM could also influence HLA affinity or cathepsin cleavage.

There are several limitations to this study. The number of included patients was quite low. Most importantly the findings *in silico* study needs to be further validated *in vitro*. In this study, the controls were restricted to OIND patients. To address whether the proposed mechanism drives the intrathecal synthesis of oligoclonal IgG in MS, MS patients without evidence of this phenomenon could be informative. Such patients are however rare, and can be hard to identify as the absence of two or more OCB bands by routine methods not necessarily rules out intrathecal synthesis of oligoclonal Ig (85–88). Moreover, the proposed hypothesis does not exclude that individuals without intrathecal synthesis of oligoclonal IgG have a repertoire of idiotope-matched T–B cell pairs, but rather that a break of immune tolerance against self-IgG leads to dysregulated idiotope-driven T–B cell collaboration (19). This corresponds to other candidate autoantigens in MS, as myelin-specific T cells in the blood of healthy individuals are a frequent finding (89). Moreover, T cell responses against self-IgG is not unique for MS, but have previously been shown in patients with other inflammatory diseases such as systemic lupus erythematosus (90–92), granulomatosis with polyangiitis (Wegener’s granulomatous) (93), and rheumatoid arthritis (94).

## Conclusion

The overwhelming complexity of the immune repertoire calls for novel approaches to chart the interactions between immune receptors. This is the first work combining high-throughput sequencing of the IGHV transcriptome with *in silico* predictions analysis for T cell activation in a human disease. We predict that the three proposed prerequisites (successful endosomal processing, high HLA class II affinity and sufficiently rare TCEM) for idiotope-driven T–B cell collaboration are likely to occur in the CDR3 region in the CSF of MS patients, with as many as 42% of the highly transcribed IGHV sequences possess at least one segment with these features.

## Ethics Statement

All participants provided written informed consent for participation. The study was approved by the Committee for Research Ethics in the South-Eastern Norwegian Healthy Authority (REK Sør-Øst S-04143a), the Norwegian Social Science Data Services (no. 11069) and the review boards at AHUS and OUS.

## Author Contributions

RH contributed with dataset preparations, statistical designs, analysis and interpretation of the data, and drafting the manuscript. AL contributed by sample acquisition, interpreting the data, and writing the manuscript. JJ contributed with collection and preparation of samples and provided the immunosequence patient database and revising the manuscript. JH contributed by interpreting the data and revising the manuscript. JB contributed by designing and performing statistical experiments, the interpretation of these and revising the manuscript. BB contributed by developing the concept and design of the study, as well as revising the manuscript. HR contributed by design of immunosequencing techniques as well as revising the manuscript. RB contributed by designing the bioinformatics algorithms, preparing datasets, interpreting the data and revising the manuscript. TH contributed by designing the study, interpreting the data, and writing the manuscript.

## Conflict of Interest Statement

RB and JH hold equity in EigenBio, the company responsible for designing the bioinformatics models used in this project. HR is an equity holder in Adaptive Biotechnologies. The two companies are independent. All other authors declare that the research was conducted in the absence of any commercial or financial relationships that could be construed as a potential conflict of interest.

## Abbreviations

APC, antigen-presenting cell; BCR, B cell receptor; CDR, complementarity determining region; mAb, monoclonal antibody; c/mTEC, cortical/medullary thymic epithelial cell; CI, confidence interval; CNS, central nervous system; CSF, cerebrospinal fluid; FC, Frequency class; FW, framework; HLA, human leukocyte antigen; IC_50_, the half maximal inhibitory concentration; IgG, immunoglobulin G; IGHV, immunoglobulin heavy variable; MHC, major histocompatibility complex; MS, multiple sclerosis; OIND, other inflammatory neurological disease; TCEM, T cell-exposed motif.
