# Supplementary material for: In Silico Prediction Analysis of Idiotope-Driven T–B Cell Collaboration in Multiple Sclerosis
Source: Front Immunol. 2017 Oct 2;8:1255. doi: 10.3389/fimmu.2017.01255 (PMC5630699; doi:10.3389/fimmu.2017.01255)
Supplement: Supplementary file 1 [file Data_Sheet_1.PDF]

Supplementary Material

In silico prediction analysis of idiotope-driven T-B cell collaboration in multiple sclerosis

Rune A. Høglund*[1,2], Andreas Lossius[1,3], Jorunn N. Johansen[3], Jane Homan[4], Jūratė Šaltytė Benth'[5], Harlan Robins[6], Bjarne Bogen[2,3,7], Robert Bremel[4†] and Trygve Holmøy[1,2†]

***Correspondence:**
Rune A. Høglund, MD
Clinical Neuroscience group, Department of Neurology, Akershus University Hospital, Postbox 1000, N-1478 Lørenskog, Norway.
Phone: +47.67963957; E-mail: r.a.hoglund@medisin.uio.no

S1 - Subject characteristics at time of sample collections

ID	Sex	Age	Diagnosis	Disease duration (months)[a]	Previous or ongoing treatment	Number of relapses	Time since relapse (months)	OCB[b]	CSF cell count[c]	Albumin ratio	IgG index
**MS-1**	F	29	RR-MS[d]	14	None	1	14	+	3	4	0.85
**MS-2**	F	33	RR-MS	156	IFN β 1a (48 months)	5	60	+	1	5.1	0.81
**MS-3**	F	36	RR-MS	66	GA (27 months)	4	33	+	3	3.8	1.7
**MS-4**	F	63	RR-MS	23	IFN β 1b (2 months)	1	23	+	1	3.6	0.68
**MS-5**	F	27	RR-MS	6	None	4	4	+	2	3.8	1.2
**MS-6**	F	33	RR-MS[d]	23	None	1	23	+	3	3.5	0.55
**MS-7**	M	33	RR-MS	48	None	3	3	+	10	17	0.9
**MS-8**	F	42	RR-MS	64	None	2	20	+	1	5.5	0.94
**MS-9**	F	34	RR-MS	2	None	1	2	+	25	6	2.93
**MS-10**	F	42	RR-MS	35	None	2	18	+	11	3.2	1.06
**MS-11**	F	20	RR-MS	13	None	2	1	+	10*	4.8*	1.2*
**OIND-1**	M	45	Aseptic meningitis	1	None	N/A	N/A	-	17	8	0.48
**OIND-2**	M	28	Aseptic meningoencephalitis	0	None	N/A	N/A	-	40	18.7	0.64
**OIND-3**	M	39	Neurosarcoidosis	13	None	N/A	N/A	-	75	31	0.65
**OIND-4**	M	42	Polyradiculitis	7	None	N/A	N/A	+	10	8	0.88
**OIND-5**	F	45	Aseptic meningitis	0	None	N/A	N/A	-	14	3	0.48
**OIND-6**	M	48	Neurosarcoidosis	16	None	N/A	N/A	-	20	26	0.46

F: female; M: male; RR-MS: relapse-remitting multiple sclerosis; OIND: other inflammatory neurological disease; IFN: interferon; GA: glatiramer acetate
[a] since first symptom, [b] Oligoclonal bands (OCB) positive indicates presence of >2 CSF specific IgG bands on isoelectric focusing, [c] number of mononuclear cells per microliter CSF, [d] Patients MS-1 and MS-6 had clinically isolated syndrome at inclusion, but later developed definite RR-MS. *values are from a 1 month prior sampling. The sample from which we acquired cells for sequencing was stopped after 2 mL due to accidental bleeding.

S2 – List of human leukocyte antigen (HLA) alleles included in the prediction analyses

HLA class I		HLA class II		
A	B	DRB	DP	DQ
01:01	07:02	1*01:01	DPA1*01:03-DPB1*02:01	DQA1*01:01-DQB1*05:01
02:01	08:01	1*03:01	DPA1*01:03-DPB1*04:02	DQA1*01:02-DQB1*06:02
02:02	15:01	1*04:01	DPA1*01:03-DPB1*04:01	DQA1*03:01-DQB1*03:02
02:03	15:03	1*04:04	DPA1*02:01-DPB1*01:01	DQA1*04:01-DQB1*04:02
02:06	18:01	1*04:05	DPA1*02:01-DPB1*05:01	DQA1*05:01-DQB1*02:01
03:01	27:05	1*07:01	DPA1*03:01-DPB1*04:02	DQA1*05:01-DQB1*03:01
11:01	35:01	1*08:02		
23:01	40:01	1*09:01		
24:02	40:02	1*11:01		
24:03	44:02	1*12:01		
26:01	44:01	1*13:02		
29:02	51:01	1*15:01		
30:01	53:01	3*01:01		
30:02	54:01	3*02:02		
31:01	57:01	4*01:01		
32:01	58:01	5*01:01		
33:01				
68:01				
68:02				
69:01				

S3 – IGHV characteristics

	Unique IGHV (N)		IGHV family usage (%) CSF							Blood						
ID	CSF	Blood	1	2	3	4	5	6	7	1	2	3	4	5	6	7
MS-1	98	238,951	30.6	3.1	33.7	31.6	0.0	1.0	0.0	25.3	5.2	48.2	12.0	6.8	0.8	1.7
MS-2	241	264,483	38.6	5.4	40.7	10.8	3.7	0.4	0.4	22.5	3.4	54.2	14.3	4.9	0.7	0.0
MS-3	417	118,547	30.5	9.8	13.2	45.8	0.0	0.5	0.2	19.9	4.2	46.1	20.4	8.3	1.1	0.0
MS-4	109	235,356	6.4	9.2	39.4	37.6	5.5	1.8	0.0	27.2	3.9	41.1	13.7	13.6	0.5	0.0
MS-5	1,409	157,163	4.1	10.1	13.8	70.7	0.6	0.4	0.4	29.1	4.8	40.5	13.5	9.8	0.6	1.7
MS-6	3,409	110,489	23.3	3.8	46.3	18.5	5.8	2.2	0.0	24.9	4.5	43.0	16.3	9.8	1.5	0.1
MS-7	2,753	117,643	22.8	5.5	31.4	34.1	4.2	2.0	0.0	21.5	6.0	42.8	21.5	7.1	1.0	0.1
MS-8	1,432	35,456	3.1	5.0	12.2	79.1	0.3	0.1	0.3	27.1	5.1	46.8	12.5	5.7	1.1	1.6
MS-9	14,413	158,631	2.9	9.0	13.2	74.4	0.3	0.1	0.0	22.0	4.4	46.8	16.6	8.0	0.6	1.6
MS-10	617	105,514	10.7	5.5	38.9	40.7	3.9	0.3	0.0	21.7	3.4	51.4	16.0	6.3	1.2	0.0
MS-11	658	8,921	12.5	6.1	39.7	34.3	6.1	0.3	1.1	9.4	1.8	58.2	16.0	11.9	2.0	0.7
OIND-1	10,247	-	15.1	3.1	53.4	21.9	5.4	1.1	0.0	-	-	-	-	-	-	-
OIND-2	4,115	-	20.6	3.5	55.1	15.3	3.7	0.7	1.0	-	-	-	-	-	-	-
OIND-3	8,283	-	20.8	3.4	47.1	22.7	5.2	0.7	0.1	-	-	-	-	-	-	-
OIND-4	5,091	5,978	4.8	2.6	22.5	67.4	2.0	0.7	0.0	19.9	4.9	52.0	15.2	6.2	1.7	0.1
OIND-5	266	122,444	14.7	2.3	48.9	18.4	15.0	0.4	0.4	21.6	3.9	51.2	14.4	8.0	0.9	0.0
OIND-6	6,831	72,955	17.1	3.2	51.3	22.4	5.4	0.5	0.0	28.8	3.7	44.8	15.4	6.5	0.8	0.0

S4 – Cathepsin cleavage of IGHV transcripts

		Cathepsin S			Cathepsin L			Cathepsin B		
	Number	Mean	LCI	UCI	Mean	LCI	UCI	Mean	LCI	UCI
IGHV 1	7921	2.63	2.60	2.66	3.83	3.80	3.86	3.08	3.04	3.11
IGHV 2	3035	2.39	2.34	2.44	3.52	3.47	3.58	3.97	3.91	4.02
IGHV 3	21865	1.54	1.52	1.56	4.78	4.76	4.80	3.04	3.02	3.06
IGHV 4	24966	3.80	3.78	3.82	6.25	6.24	6.27	3.18	3.16	3.20
IGHV 5	2098	3.40	3.34	3.46	7.41	7.35	7.47	3.50	3.43	3.56
IGHV 6	430	2.41	2.28	2.54	4.59	4.45	4.72	2.26	2.11	2.41
IGHV 7	74	2.76	2.44	3.07	5.04	4.71	5.37	4.89	4.53	5.25

L/UCI – Lower and upper 95% confidence interval

S5 – Distribution of cleavage probabilities

Supplementary Figure S5

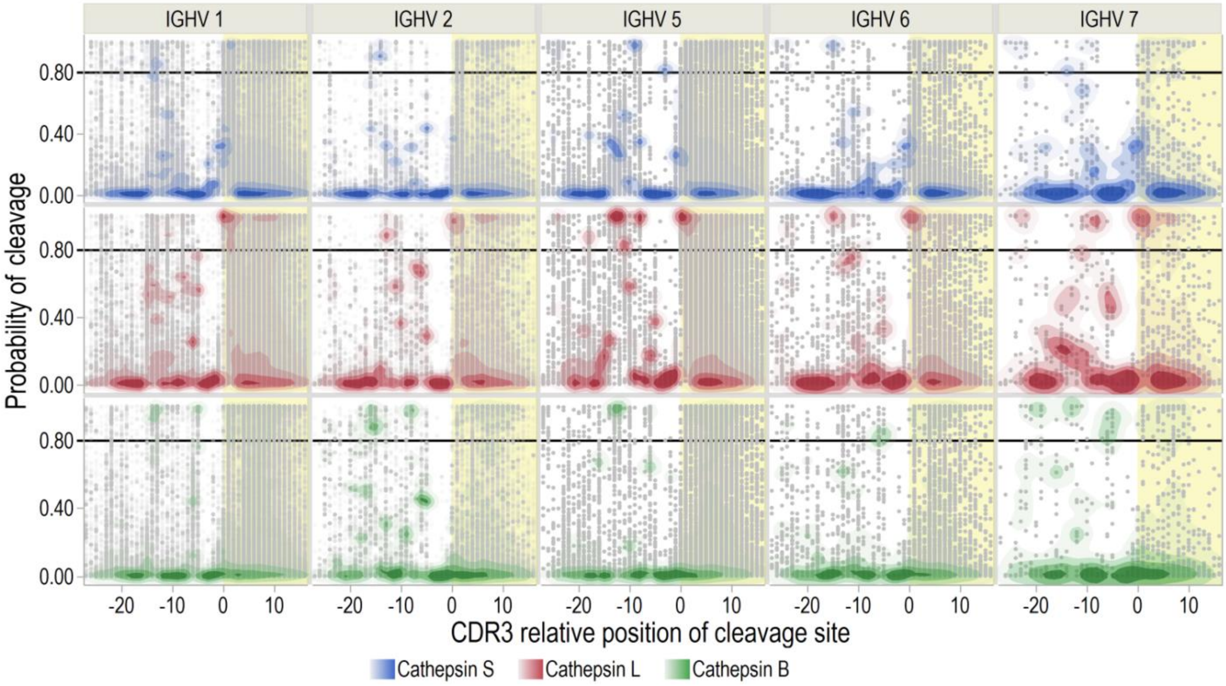

Cathepsin cleavage sites in immunoglobulin heavy variable (IGHV) transcripts were predicted with neural-net models. The distribution of predicted probabilities for cleavage (range 0-1) for Cathepsin S, L and B are shown for IGHV families 1, 2 and 5-7. The cut-off lines are set at 0.8, above which is considered a high probability for cleavage. The complementarity determining region (CDR) 3 region is marked with yellow shading and the CDR3 relative position is aligned with the cleavage site at P1-P1'.

CDR3 is marked with shading. Cathepsin L shows high predicted probability of cleavage at the CDR3 start for all IGHV families. All cathepsins have predicted several hotspots in the framework (FW) 3 region.

S6 - IGHV fragment affinities for HLA class I and II molecules

Supplementary Figure S6

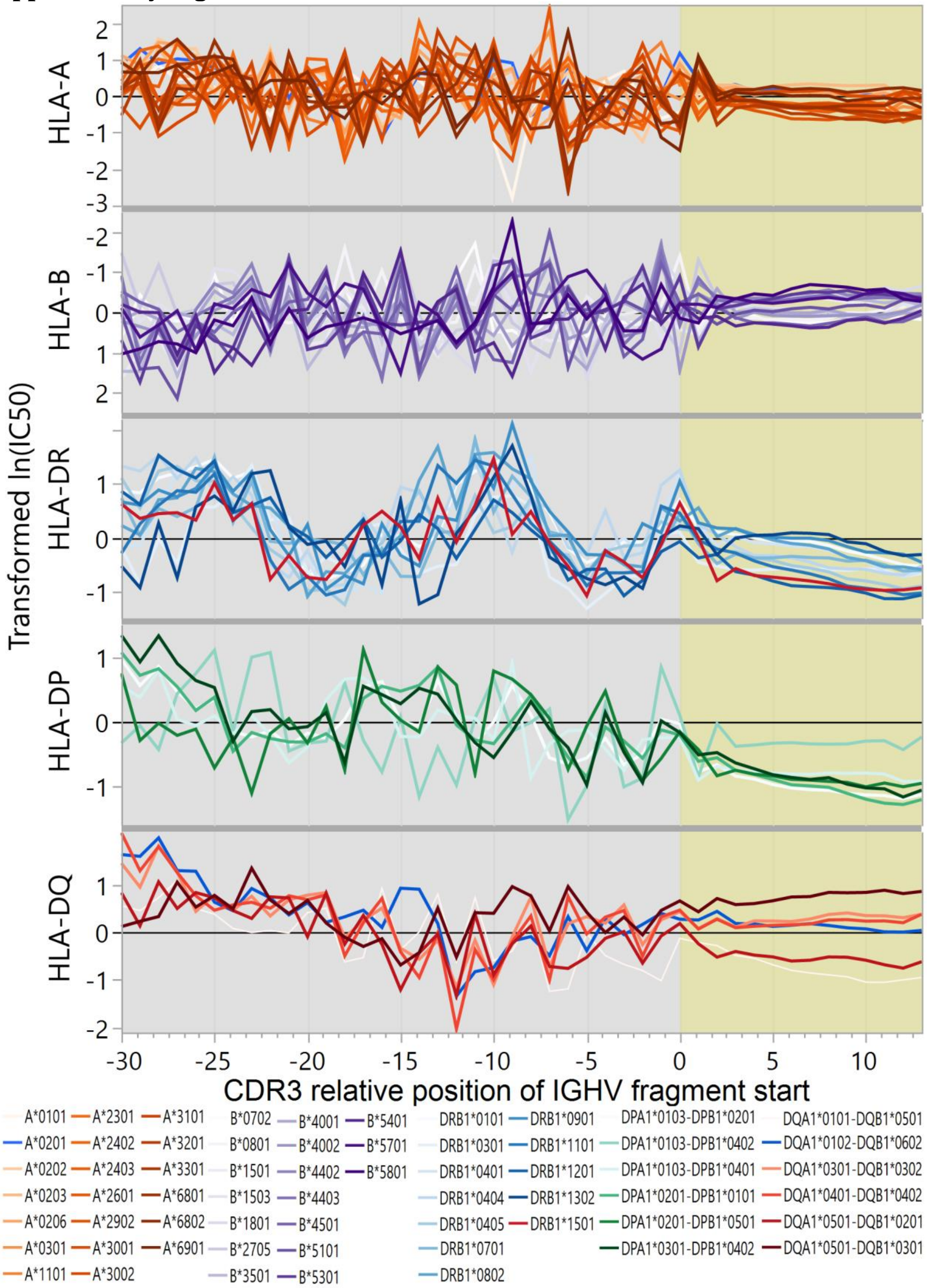

Binding affinities of CSF IGHV fragments from all patients were predicted for HLA molecules listed in Supplementary Materials S2. Results are presented as mean Johnson SI standardized values of ln (IC_{50}) calculated for each CDR3 relative position (N-terminus of a 15-mer in the case of HLA class II and a 9-mer in the case of HLA class I), bringing the different predictions to a comparable scale. Low values indicate higher affinity. Yellow shade indicates the CDR3 region.

S7 - Mean ln (IC_{50}) affinities for selected HLA molecules

Supplementary Figure S7

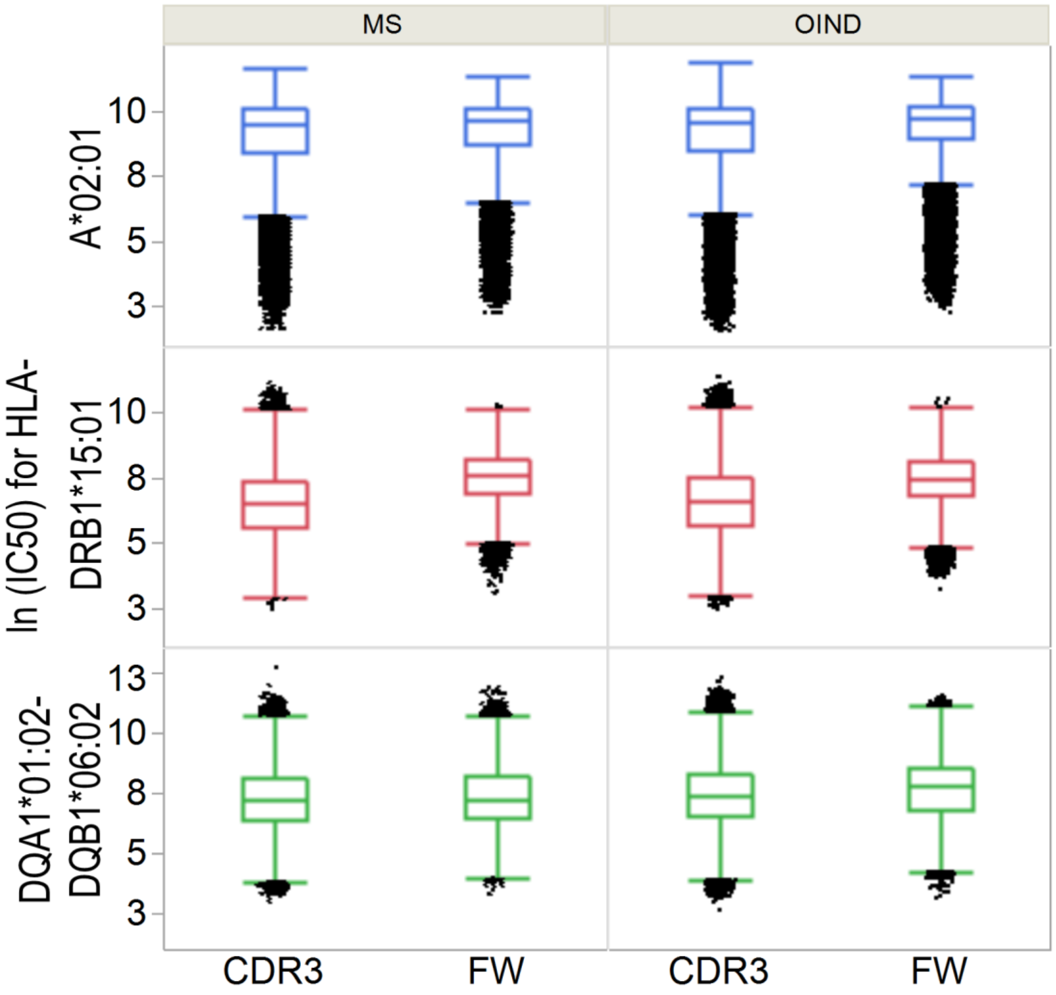

Binding affinities of IGHV fragments were predicted for HLA-A*02:01, HLA-DRB1*15:01 and HLA-DQA1*01:02-DQB1*06:02 with neural-net models. We compared the CDR3 vs FW3 region by splitting the transcripts at CDR3 relative position -7. Mean predicted ln (IC_{50}) by region (CDR3 and FW3) and by disease are shown as outlier box plots with whiskers covering 1st and 3rd quartile -/+ 1.5*(interquartile range). Table S7 shows the adjusted means used for statistical testing.

Supplementary Table S7

		HLA DRB1*15:01	HLA DQA1*01:02-DQB1*06:02	HLA A*02:01
**MS**		ICC (%)	ICC (%)	ICC (%)
	Patient-level	-	4.38	-
	CDR3 relative position	3.76	1.74	1.11
	transcript clone-level	-	-	-
	Mean difference	0.96	0.07	-0.21
	LCI	0.95	0.07	-0.22
	UCI	0.97	0.08	-0.2
	p-value	<0.001	<0.001	<0.001
		Adjusted for cluster effect: CDR3 relative position	Unadjusted for cluster effect	Adjusted for cluster effect: CDR3 relative position
**OIND**		ICC (%)	ICC (%)	ICC (%)
	Patient-level	-	-	0.7
	CDR3 relative position	3.1	2.49	-
	transcript clone-level	-	-	-
	Mean difference	0.86	-0.75	0.21
	LCI	0.85	-0.76	0.20
	UCI	0.86	-0.74	0.21
	p-value	<0.001	<0.001	<0.001
		Unadjusted for cluster effect	Adjusted for cluster effect CDR3 relative position	Unadjusted for cluster effect

ICC – Intra class correlation, L/UCI – Lower and Upper Confidence interval

S8 – Statistical tests of affinity for disease associated HLA molecules

Supplementary Table S8a – Intra class correlation on patient level

DQA1_0102-DQB1_0602		DRB1_1501		A_0201	
RelPos	ICC (%)	RelPos	ICC (%)	RelPos	ICC (%)
-7	21,0	-7	11,2	-7	28,0
-6	20,0	-6	9,3	-6	6,7
-5	19,8	-5	10,4	-5	18,4
-4	7,9	-4	17,8	-4	13,5
-3	23,6	-3	9,4	-3	20,1
-2	8,1	-2	9,7	-2	25,3
-1	15,9	-1	9,2	-1	14,6
0	8,2	0	9,9	0	5,4
1	6,5	1	7,7	1	14,8
2	19,6	2	13,8	2	10,9
3	10,1	3	8,9	3	12,4
4	14,2	4	7,0	4	9,3
5	10,6	5	10,1	5	14,9
6	15,9	6	12,9	6	7,8
7	19,3	7	15,5	7	11,6
8	13,8	8	16,3	8	11,1
9	20,6	9	13,8	9	15,4
10	14,1	10	14,7	10	26,3
11	19,2	11	16,2	11	17,0
12	12,3	12	13,2	12	8,4
13	13,6	13	12,3	13	14,0
14	12,8	14	14,6	14	15,3
15	15,3	15	17,0	15	12,7
16	14,6	16	20,2	16	11,4
17	15,1	17	18,2	17	17,2
18	13,4	18	18,1	18	14,7
19	20,1	19	22,9	19	21,3
20	20,7	20	24,2	20	22,0
21	18,9	21	22,7	21	21,4
22	20,0	22	20,6	22	22,2
23	38,5	23	36,6	23	25,8
24	21,9	24	17,5	24	34,0
25	38,2	25	31,5	25	17,9
26	19,7	26	22,2	26	36,7
27	43,3	27	44,1	27	17,7
28	13,5	28	35,0	28	32,2
29	70,1	29	52,4	29	59,2
30	0,0	30	66,0	30	0,0

ICC=Intra class correlation

Supplementary Table S8b – Mean difference in predicted affinity (ln (IC50) for HLA-DRB1*15:01 for IGHV4 fragments compared to other IGHV families

**DRB1_1501**	**IGHV_family 4 vs others**				Benj.-Hoch.	Estimated
RelPos	Mean diff	p-value	LCI	UCI	**SIGN adj**	**SIGN**
-7	-0,122	0,000	-0,135	-0,011	*	*
-6	-0,104	0,000	-0,118	-0,090	*	*
-5	-0,066	0,000	-0,079	-0,053	*	*
-4	0,006	0,467	-0,011	0,023		
-3	0,022	0,028	0,002	0,041	*	*
-2	-0,079	0,000	-0,098	-0,059	*	*
-1	-0,136	0,000	-0,158	-0,114	*	*
0	-0,074	0,000	-0,096	-0,053	*	*
1	-0,065	0,000	-0,090	-0,041	*	*
2	-0,128	0,000	-0,152	-0,104	*	*
3	-0,034	0,006	-0,058	-0,010	*	*
4	-0,007	0,564	-0,031	0,017		
5	-0,043	0,000	-0,067	-0,020	*	*
6	-0,064	0,000	-0,088	-0,041	*	*
7	-0,113	0,000	-0,136	-0,090	*	*
8	-0,095	0,000	-0,118	-0,073	*	*
9	-0,107	0,000	-0,129	-0,084	*	*
10	-0,096	0,000	-0,117	-0,074	*	*
11	-0,098	0,000	-0,121	-0,074	*	*
12	-0,076	0,000	-0,101	-0,051	*	*
13	-0,042	0,001	-0,067	-0,016	*	*
14	0,046	0,001	0,019	0,073	*	*
15	-0,044	0,003	-0,073	-0,015	*	*
16	-0,084	0,000	-0,118	-0,050	*	*
17	-0,108	0,000	-0,149	-0,067	*	*
18	-0,069	0,010	-0,122	-0,017	*	*
19	-0,010	0,761	-0,071	0,052		
20	0,158	0,000	0,080	0,236	*	*
21	0,044	0,504	-0,085	0,173		
22	0,192	0,037	0,012	0,373	*	*
23	0,405	0,002	0,150	0,660	*	*
24	-0,622	0,067	-1,289	0,044	*	
25	-0,112	0,714	-0,711	0,487		
26	0,491	0,195	-0,252	1,235		
27	-0,137	0,841	-1,476	1,203		

LCI=Lower confidence interval. UCI=upper confidence interval. ICC=Intra class correlation
Black stars indicate significant differences. Red stars indicate significant differences after correction for multiple testing (20% FDR, Benjamini-Hochberg)

Supplementary Table S8c – Mean difference in predicted affinity (ln (IC50) for HLA-DQA1*01:02-DQB1*06:02 for IGHV4 fragments compared to other IGHV families

DQA1_0102-DQB1_0602	IGHV_family 4 vs others				Benj.-Hoch.	Estimated
RelPos	Mean diff	p-value	LCI	UCI	SIGN adj	SIGN
-7	-0,414	0,000	-0,432	-0,397	*	*
-6	-0,083	0,000	-0,103	-0,062	*	*
-5	-0,163	0,000	-0,186	-0,140	*	*
-4	-0,071	0,000	-0,094	-0,049	*	*
-3	-0,069	0,000	-0,094	-0,045	*	*
-2	0,031	0,011	0,007	0,055	*	*
-1	0,059	0,000	0,036	0,083	*	*
0	0,099	0,000	0,077	0,120	*	*
1	-0,001	0,944	-0,024	0,023		
2	0,063	0,000	0,039	0,086	*	*
3	0,130	0,000	0,108	0,152	*	*
4	0,048	0,000	0,026	0,070	*	*
5	0,067	0,000	0,046	0,088	*	*
6	0,156	0,000	0,134	0,178	*	*
7	0,010	0,343	-0,011	0,031		
8	0,056	0,000	0,034	0,078	*	*
9	0,108	0,000	0,085	0,131	*	*
10	-0,062	0,000	-0,087	-0,037	*	*
11	-0,041	0,003	-0,068	-0,014	*	*
12	0,054	0,000	0,025	0,084	*	*
13	0,040	0,014	0,008	0,071	*	*
14	-0,056	0,002	-0,091	-0,020	*	*
15	0,024	0,240	-0,016	0,063		
16	0,063	0,008	0,017	0,110	*	*
17	-0,057	0,067	-0,118	0,004	*	
18	-0,047	0,199	-0,118	0,025		
19	-0,156	0,000	-0,239	-0,073	*	*
20	-0,421	0,000	-0,527	-0,316	*	*
21	-0,143	0,083	-0,304	0,019	*	
22	-0,451	0,000	-0,689	-0,213	*	*
23	-0,601	0,000	-0,908	-0,294	*	*
24	0,842	0,033	0,066	1,618	*	*
25	0,286	0,487	-0,521	1,092		
26	-0,702	0,128	-1,607	0,203	*	
27	0,195	0,814	-1,426	1,816		

LCI=Lower confidence interval. UCI=upper confidence interval. ICC=Intra class correlation
Black stars indicate significant differences. Red stars indicate significant differences after correction for multiple testing (20% FDR, Benjamini-Hochberg)

Supplementary Table S8d – Mean difference in predicted affinity (ln (IC50) for HLA-A*01:02 for IGHV4 fragments compared to other IGHV families

A_0201	IGHV_family 4 vs others				Benj.-Hoch.	Estimated
RelPos	Mean diff	p-value	LCI	UCI	SIGN adj	SIGN
-7	-0,514	0,000	-0,521	-0,507	*	*
-6	-0,059	0,000	-0,067	-0,052	*	*
-5	-0,203	0,000	-0,222	-0,185	*	*
-4	-0,104	0,000	-0,132	-0,076	*	*
-3	0,003	0,860	-0,026	0,032		
-2	-0,077	0,000	-0,109	-0,045	*	*
-1	-0,009	0,412	-0,029	0,012		
0	0,054	0,000	0,042	0,066	*	*
1	0,039	0,000	0,024	0,055	*	*
2	0,060	0,000	0,037	0,083	*	*
3	0,116	0,000	0,090	0,142	*	*
4	0,013	0,373	-0,016	0,042		
5	-0,013	0,436	-0,046	0,020		
6	-0,043	0,007	-0,075	-0,012	*	*
7	-0,052	0,003	-0,086	-0,018	*	*
8	0,101	0,000	0,064	0,138	*	*
9	-0,051	0,019	-0,094	-0,009	*	*
10	-0,363	0,000	-0,408	-0,318	*	*
11	-0,181	0,000	-0,228	-0,134	*	*
12	-0,041	0,127	-0,093	0,012		
13	0,004	0,892	-0,055	0,063		
14	-0,343	0,000	-0,410	-0,275	*	*
15	-0,235	0,000	-0,308	-0,161	*	*
16	-0,024	0,567	-0,105	0,057		
17	-0,012	0,827	-0,117	0,094		
18	-0,287	0,000	-0,420	-0,154	*	*
19	-0,704	0,000	-0,867	-0,550	*	*
20	-0,008	0,936	-0,200	0,185		
21	-0,149	0,276	-0,417	0,119		
22	-0,458	0,036	-0,885	-0,030	*	*
23	0,308	0,285	-0,256	0,871		
24	0,440	0,545	-0,986	1,866		
25	0,202	0,794	-1,313	1,717		
26	-0,207	0,827	-0,063	1,649		
27	-1,440	0,426	-4,983	2,104		

LCI=Lower confidence interval. UCI=upper confidence interval. ICC=Intra class correlation
Black stars indicate significant differences. Red stars indicate significant differences after correction for multiple testing (20% FDR, Benjamini-Hochberg)

S9 – Hierarchical clustering of Z-standardized TCEM occurrences

Supplementary figure S9a - Hierarchical clustering of Z-standardized TCEMI

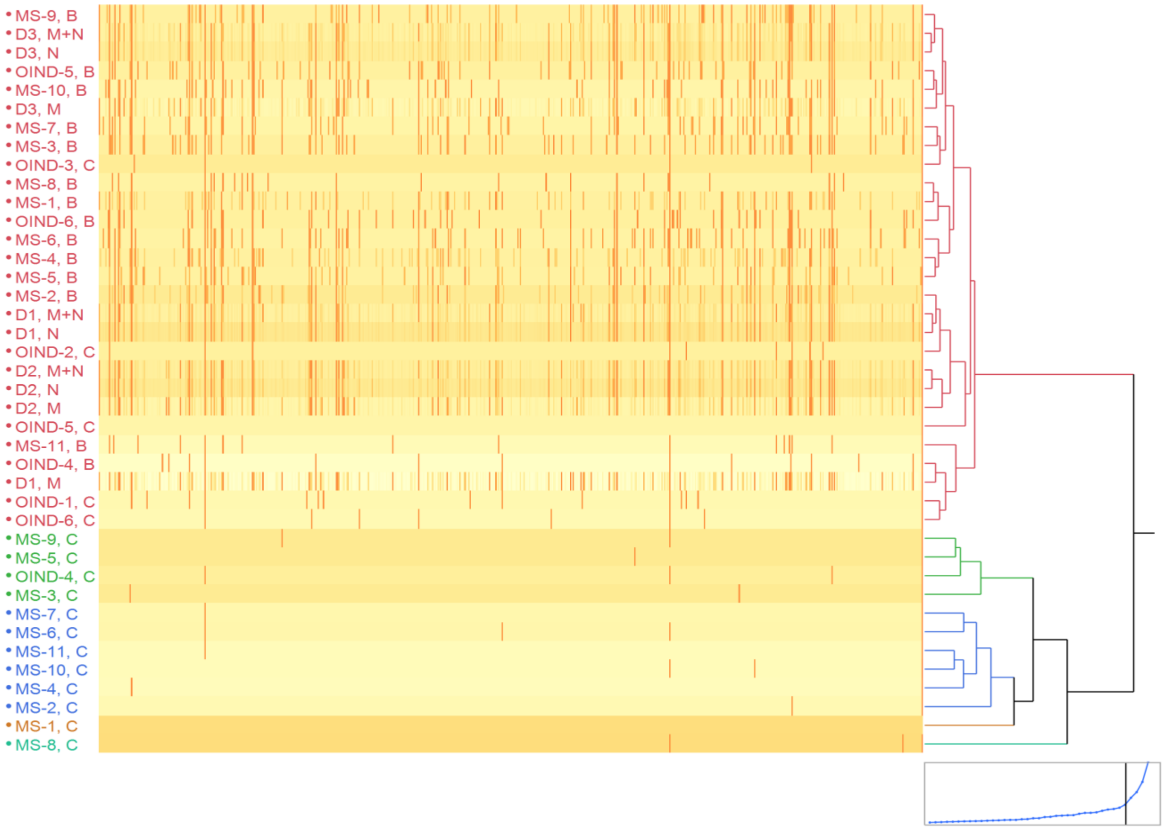

The occurrences of all TCEM (x axis) in IGHV transcripts were identified and summarized by patient and compartment in patients with MS (MS 1-11), and OIND (OIND 1-6) and three healthy individuals (D1-3). The figures S9a-c are the results of hierarchical clustering analysis with Wards' method of the Z-standardized occurrences within patient and compartment for all three TCEM patterns. N=Naïve B cells from blood, M=Mature B cells from blood. In each patient B at the end of the identification code denotes blood and C denotes CSF.

Supplementary Figure S9b - Hierarchical clustering of Z-standardized TCEMIIa

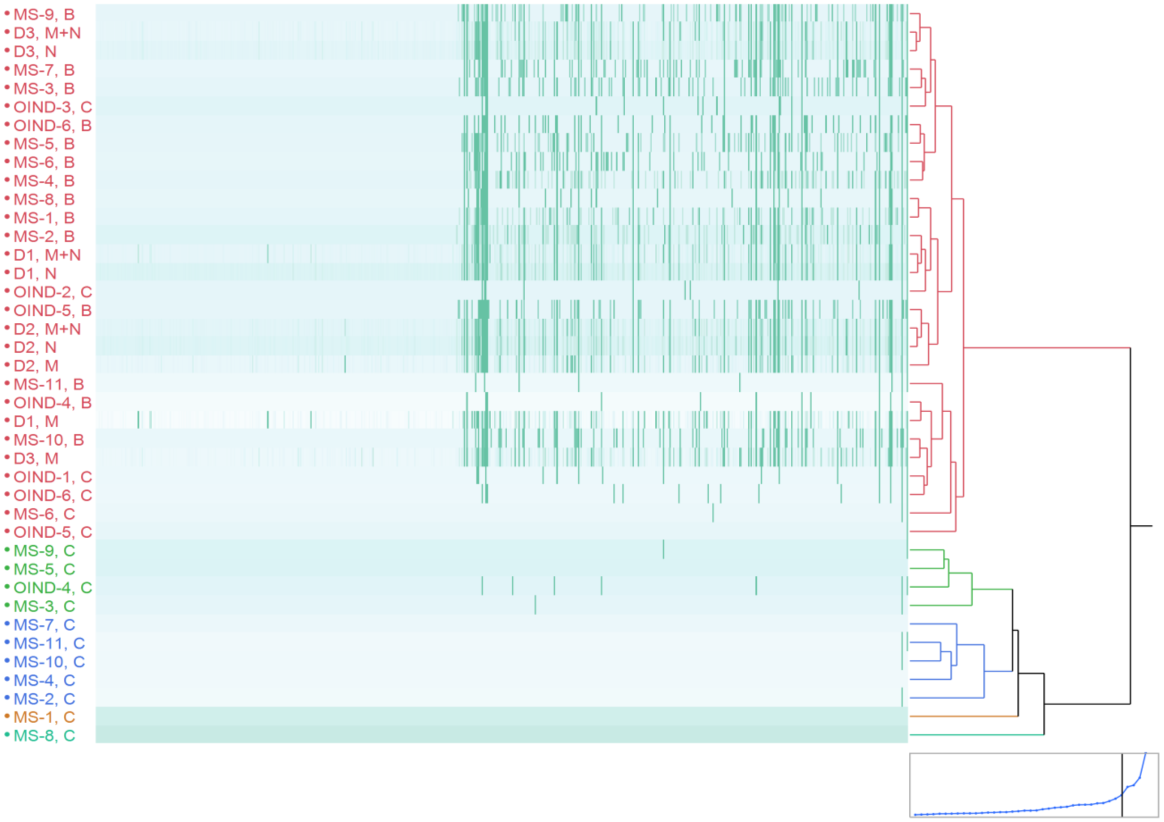

Supplementary Figure S9c - Hierarchical clustering of Z-standardized TCEMIIb

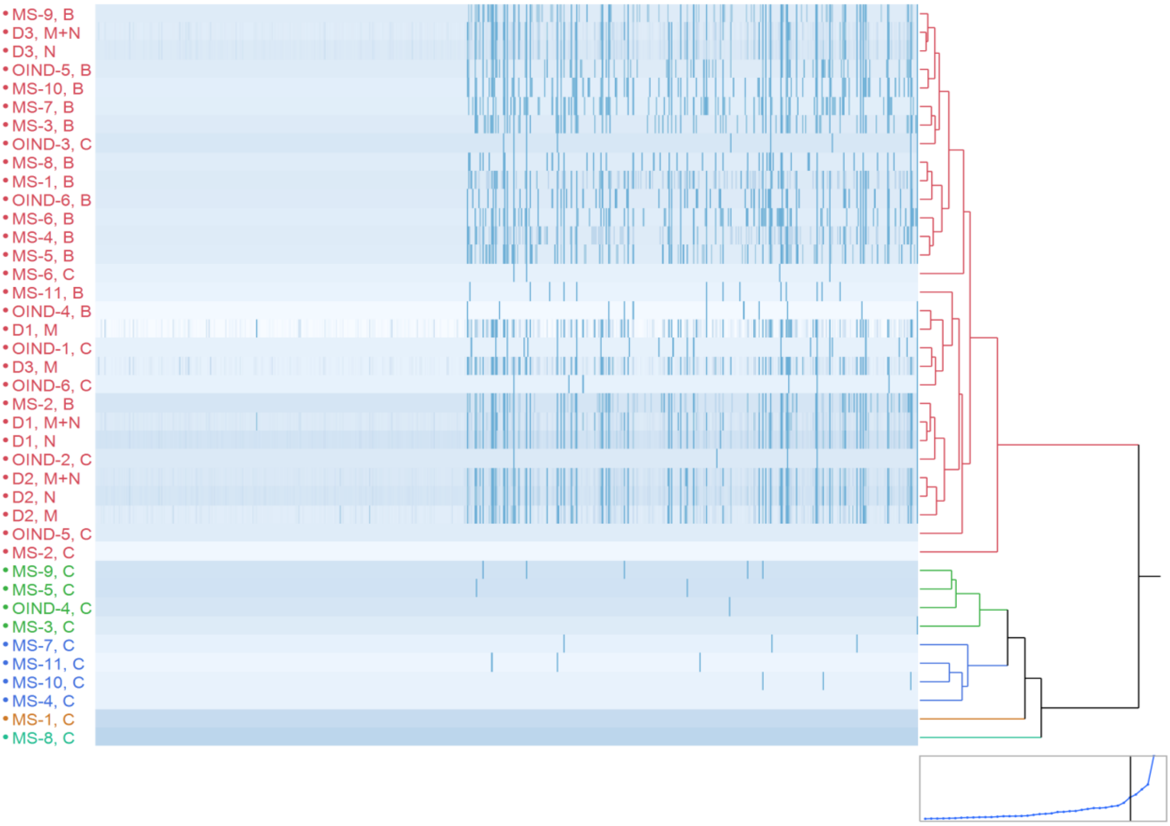

S10 – Hierarchical clustering of pairwise correlations of T cell exposed motif occurrences within each compartment and IGHV family

Supplementary Figure S10

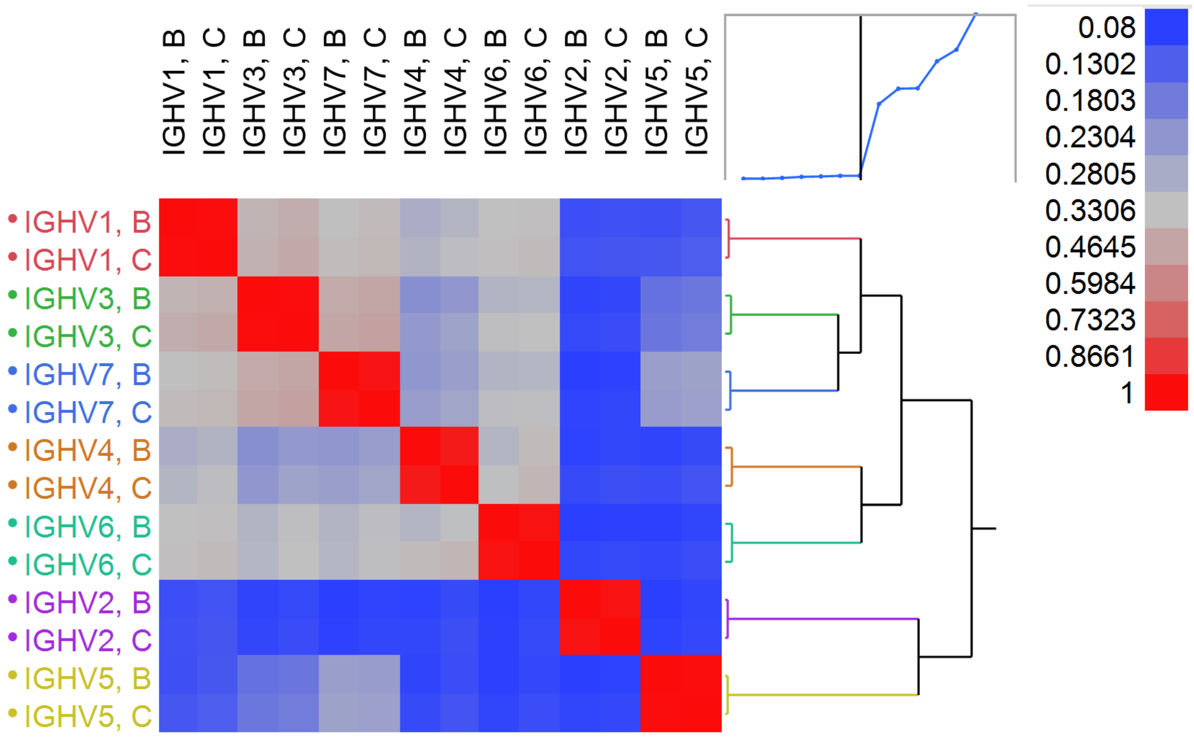

We identified all possible T cell exposed motifs (TCEM) IIa in IGHV transcripts and counted their occurrences in blood and cerebrospinal fluid (CSF) and within each IGHV family. B denotes blood- and C denotes CSF derived IGHV transcripts. Hierarchical clustering analysis with Wards' method of pairwise correlation coefficients shows that clustering occurs on IGHV family level. The pairwise metric analysis can only be computed if a TCEM is present in both samples and the observed correlations are thus not attributable to differences in sample size.

S11 – Comparison of mean FC in blood and CSF at CDR3 relative positions

Supplementary Table S11a - Mean difference of TCEMI frequency class between blood and CSF in MS patients

	**MS; Blood vs CSF**	mean(0)-mean(1)			Benj.-Hoch.	Estimated
RelPos	Mean diff	p-value	LCI	UCI	SIGN adj	SIGN
-32	-3,850	0,000	-4,492	-3,208	*	*
-31	-2,857	0,000	-3,503	-2,211	*	*
-30	-2,976	0,000	-3,416	-2,535	*	*
-29	-3,348	0,000	-3,596	-3,101	*	*
-28	-2,060	0,000	-2,215	-1,905	*	*
-27	-2,765	0,000	-2,883	-2,648	*	*
-26	-1,736	0,000	-1,835	-1,636	*	*
-25	-2,126	0,000	-2,203	-2,049	*	*
-24	-2,839	0,000	-2,902	-2,775	*	*
-23	-2,752	0,000	-2,813	-2,691	*	*
-22	-2,026	0,000	-2,078	-1,973	*	*
-21	-3,521	0,000	-3,567	-3,475	*	*
-20	-3,707	0,000	-3,754	-3,660	*	*
-19	-4,565	0,000	-4,612	-4,517	*	*
-18	-4,416	0,000	-4,462	-4,371	*	*
-17	-4,616	0,000	-4,661	-4,571	*	*
-16	-2,726	0,000	-2,768	-2,683	*	*
-15	-2,825	0,000	-2,868	-2,782	*	*
-14	-1,094	0,000	-1,134	-1,053	*	*
-13	-0,788	0,000	-0,823	-0,753	*	*
-12	-0,546	0,000	-0,582	-0,511	*	*
-11	-0,510	0,000	-0,540	-0,480	*	*
-10	-0,988	0,000	-1,023	-0,953	*	*
-9	-0,148	0,000	-0,176	-0,119	*	*
-8	-0,635	0,000	-0,668	-0,603	*	*
-7	-0,562	0,000	-0,592	-0,531	*	*
-6	-0,430	0,000	-0,465	-0,394	*	*
-5	-0,102	0,000	-0,141	-0,063	*	*
-4	-0,308	0,000	-0,350	-0,267	*	*
-3	0,141	0,000	0,102	0,181	*	*
-2	0,040	0,026	0,005	0,074	*	*
-1	-0,239	0,000	-0,272	-0,205	*	*
0	-0,854	0,000	-0,894	-0,815	*	*
1	-1,478	0,000	-1,526	-1,431	*	*
2	-2,088	0,000	-2,143	-2,033	*	*
3	-2,336	0,000	-2,397	-2,274	*	*
4	-2,251	0,000	-2,320	-2,183	*	*
5	-2,130	0,000	-2,207	-2,054	*	*
6	-2,466	0,000	-2,550	-2,383	*	*
7	-2,123	0,000	-2,214	-2,033	*	*
8	-2,218	0,000	-2,322	-2,114	*	*
9	-2,783	0,000	-2,909	-2,656	*	*
10	-3,140	0,000	-3,309	-2,971	*	*
11	-2,367	0,000	-2,587	-2,147	*	*
12	-2,887	0,000	-3,150	-2,625	*	*
13	-1,932	0,000	-2,312	-1,551	*	*
14	-4,330	0,000	-4,865	-3,796	*	*
15	-2,550	0,000	-3,360	-1,740	*	*
16	-0,559	0,252	-1,515	0,397	*	
17	-0,140	0,819	-1,339	1,059		
18	1,353	0,043	0,044	2,663	*	*
19	0,259	0,740	-1,269	1,786		
20	0,014	0,987	-1,681	1,709		
21	-1,433	0,140	-3,338	0,471	*	
22	-2,417	0,011	-4,270	-0,563	*	*
23	-2,338	0,012	-4,170	-0,506	*	*
24	-2,162	0,023	-4,021	-0,303	*	*
25	-2,441	0,007	-4,224	-0,659	*	*
26	-1,876	0,039	-3,656	-0,097	*	*
27	-1,440	0,116	-3,233	0,353	*	
28	-1,449	0,121	-3,279	0,382	*	
29	-1,054	0,349	-3,260	1,153		

LCI=Lower confidence interval. UCI=upper confidence interval. ICC=Intra class correlation
Black stars indicate significant differences. Red stars indicate significant differences after correction for multiple testing (20% FDR, Benjamini-Hochberg)

Supplementary Table S11b - Mean difference of TCEMIIa frequency class between blood and CSF in MS patients

	MS; Blood vs CSF	mean(0)-mean(1)			Benj.-Hoch.	Estimated
RelPos	Mean diff	p-value	LCI	UCI	SIGN adj	SIGN
-32	-3,289	0,000	-4,021	-2,557	*	*
-31	-2,234	0,000	-2,851	-1,616	*	*
-30	-1,511	0,000	-1,970	-1,052	*	*
-29	-3,668	0,000	-3,937	-3,400	*	*
-28	-2,333	0,000	-2,487	-2,178	*	*
-27	-1,838	0,000	-1,964	-1,713	*	*
-26	-1,645	0,000	-1,742	-1,553	*	*
-25	-1,676	0,000	-1,743	-1,608	*	*
-24	-4,679	0,000	-4,750	-4,607	*	*
-23	-4,231	0,000	-4,292	-4,170	*	*
-22	-2,875	0,000	-2,928	-2,822	*	*
-21	-4,787	0,000	-4,845	-4,729	*	*
-20	-1,834	0,000	-1,875	-1,793	*	*
-19	-4,385	0,000	-4,429	-4,340	*	*
-18	-3,327	0,000	-3,374	-3,280	*	*
-17	-2,876	0,000	-2,919	-2,832	*	*
-16	-2,534	0,000	-2,575	-2,493	*	*
-15	-1,206	0,000	-1,239	-1,173	*	*
-14	-1,261	0,000	-1,297	-1,225	*	*
-13	-0,566	0,000	-0,603	-0,528	*	*
-12	-0,514	0,000	-0,551	-0,478	*	*
-11	-1,337	0,000	-1,366	-1,309	*	*
-10	-0,545	0,000	-0,577	-0,514	*	*
-9	-0,140	0,000	-0,166	-0,114	*	*
-8	-0,214	0,000	-0,256	-0,173	*	*
-7	0,136	0,000	0,096	0,176	*	*
-6	-0,620	0,000	-0,652	-0,587	*	*
-5	-0,110	0,000	-0,144	-0,077	*	*
-4	-0,479	0,000	-0,510	-0,447	*	*
-3	-0,276	0,000	-0,311	-0,242	*	*
-2	-0,435	0,026	-0,471	-0,400	*	*
-1	-0,796	0,000	-0,833	-0,759	*	*
0	-1,253	0,000	-1,296	-1,211	*	*
1	-1,381	0,000	-1,427	-1,335	*	*
2	-1,583	0,000	-1,632	-1,534	*	*
3	-1,349	0,000	-1,399	-1,298	*	*
4	-1,342	0,000	-1,396	-1,287	*	*
5	-1,580	0,000	-1,641	-1,519	*	*
6	-2,081	0,000	-2,150	-2,012	*	*
7	-1,813	0,000	-1,893	-1,733	*	*
8	-1,923	0,000	-2,022	-1,829	*	*
9	-2,523	0,000	-2,641	-2,404	*	*
10	-1,992	0,000	-2,150	-1,833	*	*
11	-2,352	0,000	-2,556	-2,147	*	*
12	-2,304	0,000	-2,458	-2,061	*	*
13	-2,652	0,000	-3,005	-2,299	*	*
14	-3,966	0,000	-4,465	-3,468	*	*
15	-2,262	0,000	-3,023	-1,502	*	*
16	-1,865	0,000	-2,766	-0,964	*	*
17	-2,553	0,819	-3,686	-1,421		
18	-0,609	0,334	-1,845	0,626		
19	-2,626	0,000	-4,043	-1,209	*	*
20	-1,362	0,096	-2,966	0,242	*	
21	-2,670	0,004	-4,465	-0,875	*	*
22	-3,396	0,000	-5,149	-1,644	*	*
23	-1,345	0,122	-3,052	0,361	*	
24	-2,776	0,001	-4,402	-1,150	*	*
25	-1,154	0,119	-2,606	0,298	*	
26	-0,779	0,297	-2,243	0,685		
27	-1,672	0,023	-3,113	-0,230	*	*
28	-1,985	0,018	-3,628	-0,341	*	*
29	-1,542	0,129	-3,535	0,451	*	

LCI=Lower confidence interval. UCI=upper confidence interval. ICC=Intra class correlation
Black stars indicate significant differences. Red stars indicate significant differences after correction for multiple testing (20% FDR, Benjamini-Hochberg)

Supplementary Table S11c - Mean difference of TCEMIIb frequency class between blood and CSF in MS patients

	**MS; Blood vs CSF**	mean(0)-mean(1)			Benj.-Hoch.	Estimated
RelPos	Mean diff	p-value	LCI	UCI	**SIGN adj**	**SIGN**
-32	-3,289	0,000	-3,992	-2,586	*	*
-31	-2,475	0,000	-3,067	-1,883	*	*
-30	-2,094	0,000	-2,494	-1,694	*	*
-29	-4,395	0,000	-4,672	-4,117	*	*
-28	-1,738	0,000	-1,901	-1,576	*	*
-27	-2,019	0,000	-2,143	-1,896	*	*
-26	-2,498	0,000	-2,589	-2,408	*	*
-25	-1,625	0,000	-1,693	-1,557	*	*
-24	-4,417	0,000	-4,489	-4,345	*	*
-23	-3,981	0,000	-4,035	-3,927	*	*
-22	-3,309	0,000	-3,366	-3,253	*	*
-21	-5,093	0,000	-5,153	-5,033	*	*
-20	-1,681	0,000	-1,718	-1,644	*	*
-19	-4,501	0,000	-4,549	-4,452	*	*
-18	-1,346	0,000	-1,393	-1,299	*	*
-17	-2,696	0,000	-2,738	-2,655	*	*
-16	-2,884	0,000	-2,923	-2,845	*	*
-15	-1,130	0,000	-1,162	-1,098	*	*
-14	-2,290	0,000	-2,328	-2,252	*	*
-13	-0,331	0,000	-0,368	-0,294	*	*
-12	-1,248	0,000	-1,284	-1,212	*	*
-11	-1,436	0,000	-1,464	-1,407	*	*
-10	-0,453	0,000	-0,489	-0,418	*	*
-9	-0,852	0,000	-0,883	-0,821	*	*
-8	-0,136	0,000	-0,177	-0,096	*	*
-7	0,048	0,007	0,013	0,083	*	*
-6	-0,618	0,000	-0,650	-0,586	*	*
-5	0,345	0,000	0,309	0,382	*	*
-4	-0,492	0,000	-0,522	-0,462	*	*
-3	-0,642	0,000	-0,676	-0,608	*	*
-2	-0,766	0,000	-0,800	-0,732	*	*
-1	-0,870	0,000	-0,906	-0,834	*	*
0	-0,858	0,000	-0,898	-0,817	*	*
1	-1,031	0,000	-1,074	-0,988	*	*
2	-1,165	0,000	-1,210	-1,119	*	*
3	-1,082	0,000	-1,129	-1,034	*	*
4	-1,191	0,000	-1,243	-1,139	*	*
5	-1,300	0,000	-1,358	-1,242	*	*
6	-1,892	0,000	-1,957	-1,827	*	*
7	-1,568	0,000	-1,641	-1,495	*	*
8	-1,892	0,000	-1,978	-1,807	*	*
9	-1,926	0,000	-2,028	-1,823	*	*
10	-1,589	0,000	-1,722	-1,455	*	*
11	-1,840	0,000	-2,009	-1,671	*	*
12	-2,088	0,000	-2,286	-1,889	*	*
13	-1,587	0,000	-1,873	-1,299	*	*
14	-3,032	0,000	-3,438	-2,625	*	*
15	-1,574	0,000	-2,196	-0,952	*	*
16	-1,892	0,000	-2,639	-1,146	*	*
17	-1,760	0,000	-2,715	-0,805	*	*
18	-0,395	0,458	-1,437	0,648		
19	-3,145	0,000	-4,384	-1,906	*	*
20	-1,018	0,165	-2,455	0,420	*	
21	-1,919	0,234	-3,579	-0,260	*	
22	-2,848	0,001	-4,445	-1,251	*	*
23	-1,473	0,063	-3,028	0,081	*	
24	-2,702	0,000	-4,180	-1,224	*	*
25	-0,709	0,334	-2,147	0,729		
26	-0,838	0,262	-2,303	0,627	*	
27	-1,679	0,016	-3,048	-0,310	*	*
28	-1,583	0,037	-3,071	-0,095	*	*
29	-1,763	0,046	-3,495	-0,031	*	*

LCI=Lower confidence interval. UCI=upper confidence interval. ICC=Intra class correlation
Black stars indicate significant differences. Red stars indicate significant differences after correction for multiple testing (20% FDR, Benjamini-Hochberg)

Supplementary Table S11d - Mean difference of TCEMI frequency class between blood and CSF in OIND patients

	OIND; Blood vs CSF	mean(0)-mean(1)			Benj.-Hoch.	Estimated
RelPos	Mean diff	p-value	LCI	UCI	SIGN adj	SIGN
-33	-0,624	0,092	-1,350	0,102	*	
-32	-0,161	0,388	-0,526	0,205		
-31	-0,147	0,388	-0,480	0,186		
-30	-0,452	0,000	-0,688	-0,216	*	*
-29	-0,737	0,000	-0,919	-0,555	*	*
-28	-0,554	0,000	-0,675	-0,434	*	*
-27	-0,602	0,000	-0,700	-0,505	*	*
-26	-0,707	0,000	-0,797	-0,617	*	*
-25	-0,657	0,000	-0,734	-0,581	*	*
-24	-0,828	0,000	-0,892	-0,765	*	*
-23	-0,871	0,000	-0,930	-0,812	*	*
-22	-0,762	0,000	-0,813	-0,711	*	*
-21	-0,942	0,000	-0,988	-0,897	*	*
-20	-1,134	0,000	-1,179	-1,089	*	*
-19	-1,610	0,000	-1,657	-1,563	*	*
-18	-1,491	0,000	-1,537	-1,446	*	*
-17	-1,567	0,000	-1,612	-1,522	*	*
-16	-1,059	0,000	-1,101	-1,017	*	*
-15	-1,129	0,000	-1,172	-1,087	*	*
-14	-0,535	0,000	-0,574	-0,496	*	*
-13	-0,433	0,000	-0,467	-0,399	*	*
-12	-0,402	0,000	-0,436	-0,367	*	*
-11	-0,241	0,000	-0,271	-0,212	*	*
-10	-0,623	0,000	-0,658	-0,589	*	*
-9	-0,338	0,000	-0,366	-0,310	*	*
-8	-0,570	0,000	-0,602	-0,538	*	*
-7	-0,531	0,000	-0,561	-0,501	*	*
-6	-0,655	0,000	-0,690	-0,620	*	*
-5	-0,520	0,000	-0,557	-0,483	*	*
-4	-0,536	0,000	-0,575	-0,497	*	*
-3	-0,331	0,000	-0,368	-0,294	*	*
-2	-0,229	0,000	-0,261	-0,196	*	*
-1	-0,225	0,000	-0,256	-0,193	*	*
0	-0,494	0,000	-0,530	-0,457	*	*
1	-0,750	0,000	-0,794	-0,706	*	*
2	-0,952	0,000	-1,004	-0,901	*	*
3	-1,193	0,000	-1,250	-1,135	*	*
4	-1,352	0,000	-1,416	-1,288	*	*
5	-1,268	0,000	-1,339	-1,198	*	*
6	-1,212	0,000	-1,291	-1,134	*	*
7	-1,128	0,000	-1,216	-1,040	*	*
8	-1,064	0,000	-1,165	-0,963	*	*
9	-1,022	0,000	-1,145	-0,900	*	*
10	-0,847	0,000	-0,998	-0,696	*	*
11	-0,876	0,000	-1,065	-0,686	*	*
12	-0,883	0,000	-1,118	-0,649	*	*
13	-1,196	0,000	-1,482	-0,911	*	*
14	-1,540	0,000	-1,892	-1,187	*	*
15	-1,673	0,000	-2,101	-1,244	*	*
16	-2,399	0,000	-2,909	-1,888	*	*
17	-2,861	0,000	-3,485	-2,237	*	*
18	-2,872	0,000	-3,586	-2,157	*	*
19	-1,986	0,000	-2,793	-1,178	*	*
20	-1,823	0,000	-2,712	-0,933	*	*
21	-1,282	0,011	-2,272	-0,292	*	*
22	-1,440	0,009	-2,519	-0,362	*	*
23	-2,000	0,001	-3,146	-0,854	*	*
24	-1,579	0,010	-2,775	-0,383	*	*
25	-1,608	0,004	-2,693	-0,522	*	*
26	-0,314	0,596	-1,477	0,849		
27	-0,261	0,665	-1,443	0,922		
28	-0,495	0,437	-1,748	0,758		
29	-0,090	0,903	-1,542	1,361		

LCI=Lower confidence interval. UCI=upper confidence interval. ICC=Intra class correlation
Black stars indicate significant differences. Red stars indicate significant differences after correction for multiple testing (20% FDR, Benjamini-Hochberg)

Supplementary Table S11e - Mean difference of TCEMIIa frequency class between blood and CSF in OIND patients

	OIND; Blood vs CSF	mean(0)-mean(1)			Benj.-Hoch.	Estimated
RelPos	Mean diff	p-value	LCI	UCI	SIGN adj	SIGN
-32	-0,218	0,309	-0,637	0,202		
-31	-0,071	0,672	-0,401	0,259		
-30	-0,561	0,000	-0,808	-0,314	*	*
-29	-0,877	0,000	-1,088	-0,666	*	*
-28	-0,710	0,000	-0,833	-0,588	*	*
-27	-0,509	0,000	-0,613	-0,405	*	*
-26	-0,715	0,000	-0,802	-0,628	*	*
-25	-0,457	0,000	-0,524	-0,389	*	*
-24	-1,076	0,000	-1,146	-1,006	*	*
-23	-1,318	0,000	-1,377	-1,259	*	*
-22	-1,107	0,000	-1,158	-1,055	*	*
-21	-1,485	0,000	-1,542	-1,429	*	*
-20	-0,630	0,000	-0,670	-0,591	*	*
-19	-1,402	0,000	-1,447	-1,357	*	*
-18	-1,146	0,000	-1,192	-1,100	*	*
-17	-1,240	0,000	-1,282	-1,197	*	*
-16	-0,956	0,000	-0,996	-0,916	*	*
-15	-0,403	0,000	-0,435	-0,370	*	*
-14	-0,476	0,000	-0,511	-0,441	*	*
-13	-0,632	0,000	-0,669	-0,595	*	*
-12	-0,617	0,000	-0,653	-0,580	*	*
-11	-0,457	0,000	-0,485	-0,429	*	*
-10	-0,489	0,000	-0,520	-0,459	*	*
-9	-0,302	0,000	-0,328	-0,275	*	*
-8	-0,591	0,000	-0,631	-0,551	*	*
-7	-0,468	0,000	-0,505	-0,431	*	*
-6	-0,516	0,000	-0,547	-0,484	*	*
-5	-0,303	0,000	-0,334	-0,272	*	*
-4	-0,396	0,000	-0,426	-0,366	*	*
-3	-0,252	0,000	-0,285	-0,220	*	*
-2	-0,436	0,000	-0,469	-0,403	*	*
-1	-0,449	0,000	-0,484	-0,414	*	*
0	-0,616	0,000	-0,655	-0,576	*	*
1	-0,706	0,000	-0,750	-0,663	*	*
2	-0,839	0,000	-0,886	-0,792	*	*
3	-0,839	0,000	-0,886	-0,792	*	*
4	-0,823	0,000	-0,875	-0,771	*	*
5	-0,707	0,000	-0,765	-0,649	*	*
6	-0,797	0,000	-0,863	-0,731	*	*
7	-0,804	0,000	-0,883	-0,725	*	*
8	-0,790	0,000	-0,884	-0,695	*	*
9	-0,945	0,000	-1,061	-0,828	*	*
10	-1,106	0,000	-1,250	-0,961	*	*
11	-1,229	0,000	-1,480	-1,050	*	*
12	-1,626	0,000	-1,845	-1,460	*	*
13	-1,910	0,000	-2,178	-1,643	*	*
14	-2,337	0,000	-2,674	-2,000	*	*
15	-2,358	0,000	-2,767	-1,949	*	*
16	-3,288	0,000	-3,776	-2,799	*	*
17	-3,002	0,000	-3,605	-2,400	*	*
18	-2,456	0,000	-3,144	-1,769	*	*
19	-2,759	0,000	-3,545	-1,973	*	*
20	-2,323	0,000	-3,182	-1,464	*	*
21	-1,379	0,000	-2,316	-0,441	*	*
22	-2,031	0,000	-2,969	-1,092	*	*
23	-1,228	0,024	-2,293	-0,163	*	*
24	-1,328	0,014	-2,387	-0,269	*	*
25	-0,638	0,216	-1,652	0,375	*	
26	-0,426	0,405	-1,429	0,578		
27	0,124	0,823	-0,964	1,212		
28	-0,157	0,807	-1,414	1,101		
29	-0,186	0,786	-1,536	1,163		

LCI=Lower confidence interval. UCI=upper confidence interval. ICC=Intra class correlation
Black stars indicate significant differences. Red stars indicate significant differences after correction for multiple testing (20% FDR, Benjamini-Hochberg)

Supplementary Table S11f - Mean difference of TCEMIIb frequency class between blood and CSF in OIND patients

	**OIND; Blood vs CSF**	mean(0)-mean(1)			Benj.-Hoch.	Estimated
RelPos	Mean diff	p-value	LCI	UCI	**SIGN adj**	**SIGN**
-32	-0,092	0,649	-0,491	0,306		
-31	0,115	0,474	-0,200	0,430		
-30	-0,685	0,000	-0,903	-0,466	*	*
-29	-0,867	0,000	-1,086	-0,648	*	*
-28	-0,698	0,000	-0,828	-0,567	*	*
-27	-0,466	0,000	-0,570	-0,362	*	*
-26	-0,778	0,000	-0,862	-0,695	*	*
-25	-0,555	0,000	-0,620	-0,489	*	*
-24	-1,006	0,000	-1,076	-0,936	*	*
-23	-1,065	0,000	-1,118	-1,012	*	*
-22	-1,152	0,000	-1,206	-1,098	*	*
-21	-1,653	0,000	-1,711	-1,595	*	*
-20	-0,554	0,000	-0,590	-0,518	*	*
-19	-1,493	0,000	-1,541	-1,444	*	*
-18	-0,703	0,000	-0,748	-0,657	*	*
-17	-1,081	0,000	-1,122	-1,040	*	*
-16	-0,696	0,000	-0,735	-0,658	*	*
-15	-0,574	0,000	-0,605	-0,542	*	*
-14	-0,907	0,000	-0,945	-0,870	*	*
-13	-0,377	0,000	-0,414	-0,341	*	*
-12	-0,743	0,000	-0,779	-0,707	*	*
-11	-0,578	0,000	-0,605	-0,550	*	*
-10	-0,553	0,000	-0,588	-0,517	*	*
-9	-0,417	0,000	-0,447	-0,386	*	*
-8	-0,566	0,000	-0,604	-0,527	*	*
-7	-0,259	0,000	-0,291	-0,226	*	*
-6	-0,481	0,000	-0,512	-0,450	*	*
-5	-0,274	0,000	-0,309	-0,241	*	*
-4	-0,364	0,000	-0,392	-0,335	*	*
-3	-0,326	0,000	-0,358	-0,295	*	*
-2	-0,534	0,000	-0,565	-0,503	*	*
-1	-0,472	0,000	-0,506	-0,439	*	*
0	-0,535	0,000	-0,573	-0,497	*	*
1	-0,600	0,000	-0,641	-0,560	*	*
2	-0,611	0,000	-0,655	-0,568	*	*
3	-0,622	0,000	-0,668	-0,576	*	*
4	-0,668	0,000	-0,718	-0,619	*	*
5	-0,634	0,000	-0,688	-0,579	*	*
6	-0,719	0,000	-0,781	-0,657	*	*
7	-0,701	0,000	-0,773	-0,629	*	*
8	-0,613	0,000	-0,697	-0,530	*	*
9	-0,705	0,000	-0,807	-0,604	*	*
10	-0,870	0,000	-0,992	-0,748	*	*
11	-0,952	0,000	-1,101	-0,803	*	*
12	-1,341	0,000	-1,521	-1,161	*	*
13	-1,421	0,000	-1,640	-1,203	*	*
14	-1,841	0,000	-2,114	-1,568	*	*
15	-1,873	0,000	-2,208	-1,539	*	*
16	-2,479	0,000	-2,879	-2,079	*	*
17	-2,305	0,000	-2,810	-1,799	*	*
18	-2,083	0,000	-2,659	-1,507	*	*
19	-2,798	0,000	-3,472	-2,124	*	*
20	-1,725	0,000	-2,506	-0,943	*	*
21	-1,429	0,000	-2,258	-0,600	*	*
22	-1,734	0,000	-2,561	-0,907	*	*
23	-0,574	0,224	-1,500	0,352	*	
24	-1,043	0,035	-2,010	-0,076	*	*
25	0,437	0,371	-0,523	1,398		
26	-0,014	0,978	-0,981	0,953		
27	-0,198	0,711	-1,246	0,850		
28	-0,459	0,421	-1,581	0,663		
29	-0,219	0,714	-1,398	0,959		

LCI=Lower confidence interval. UCI=upper confidence interval. ICC=Intra class correlation
Black stars indicate significant differences. Red stars indicate significant differences after correction for multiple testing (20% FDR, Benjamini-Hochberg)

S12 – Comparison of mean FC in the CDR3 vs FW3

Supplementary Figure S12a –Mean frequency of T cell exposed motifs in IGHV transcripts.

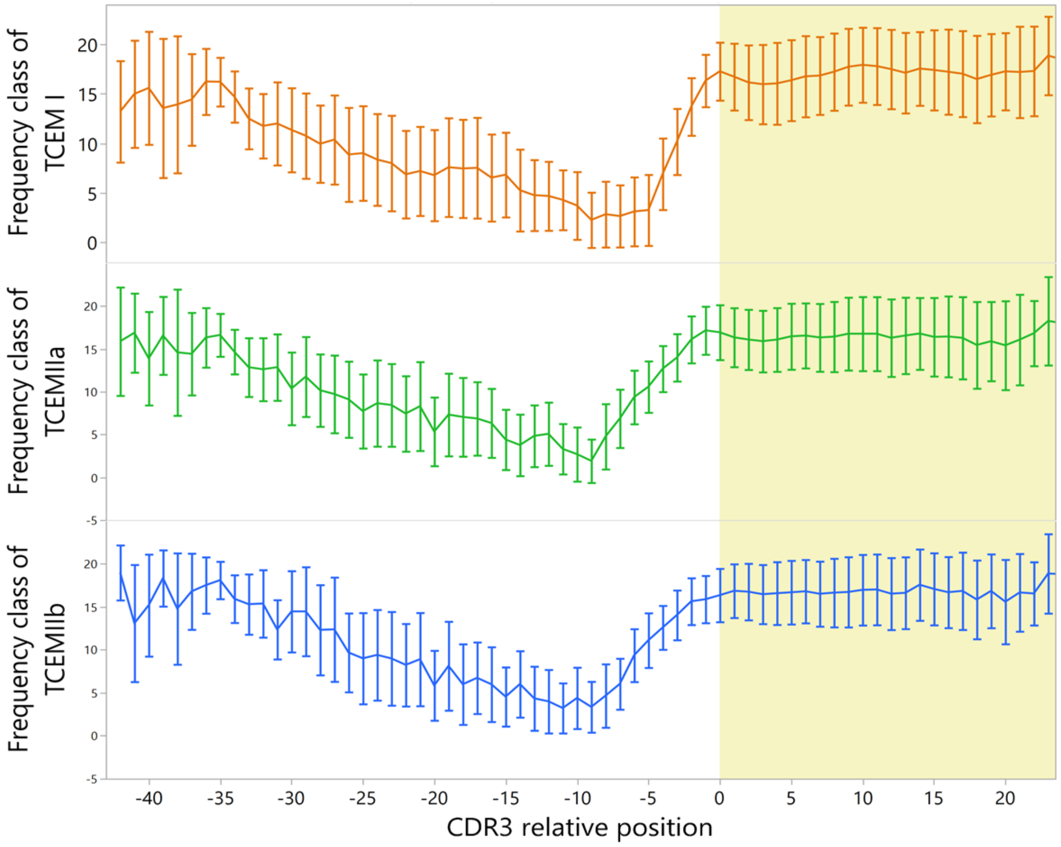

We identified all TCEM in our patients CSF IGHV fragments, and used TCEM occurrences in three healthy individuals to generate a –log2 based FC for each TCEM. Mean frequency class (FC) of all patients in the dataset is displayed for all CDR3 relative positions. Low values represent frequent TCEM. Each error bar is constructed using 1 standard deviation from the mean. The CDR3 relative position refers to first N-terminus amino acid of a 15-mer in the case of TCEM II and a 9-mer in the case of TCEM I.

Supplementary Figure S12b –Mean frequency class of CDR3 vs FW3 derived fragments in IGHV transcripts

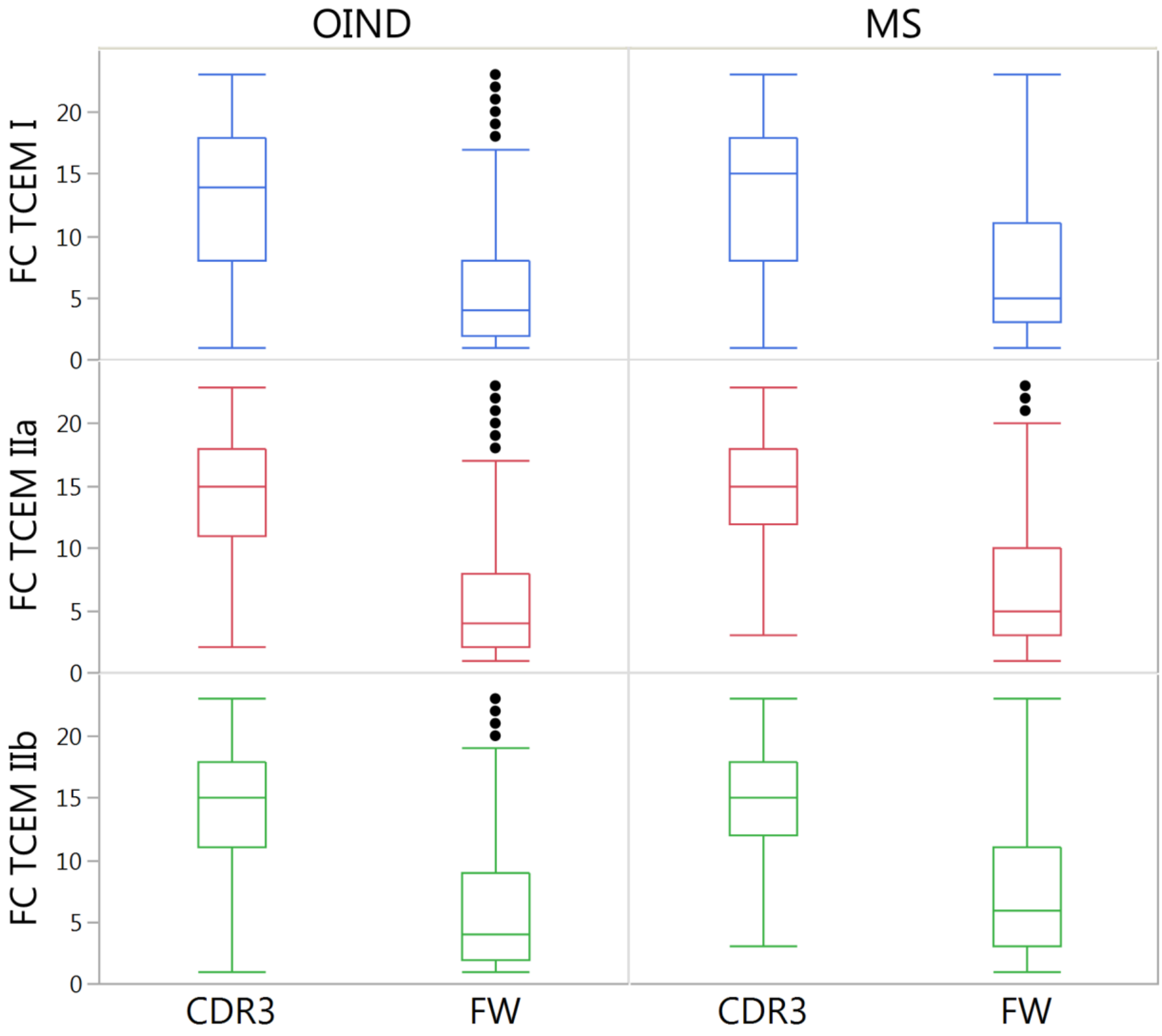

We compared the mean FC of CDR3- vs FW3-derived fragments by splitting the transcripts at CDR3 relative position -7. Mean FC by region (CDR3 and FW3) and by disease are shown as outlier box plots with whiskers covering 1st and 3rd quartile -/+ 1.5*(interquartile range). Supplementary Table S12 shows the adjusted means used for statistical testing

Supplementary Table S12 - TCEM Frequency class mean difference in FC in FW3 vs CDR3

		FC for TCEM I	FC for TCEM IIa	FC for TCEM IIb
		ICC (%)	ICC (%)	ICC (%)
	Patient-level	-	-	-
	CDR3 relative position	-	-	-
	transcript clone-level	-	-	-
**MS**	Mean difference	-5.81	-7.99	-7.46
	LCI	-5.83	-8.01	-7.49
	UCI	-5.78	-7.97	-7.44
	p-value	<0.001	<0.001	<0.001
		Unadjusted for cluster effect	Unadjusted for cluster effect	Unadjusted for cluster effect
		ICC (%)	ICC (%)	ICC (%)
	Patient-level	1.2	1.19	1.27
	CDR3 relative position	-	6.53	5.82
	transcript clone-level	-	-	-
**OIND**	Mean difference	-6.75	-8.83	-8.31
	LCI	-6.77	-8.84	-8.32
	UCI	-6.73	-8.81	-8.29
	p-value	<0.001	<0.001	<0.001
		Unadjusted for cluster effect	Unadjusted for cluster effect	Unadjusted for cluster effect

ICC – Intra class correlation, L/UCI – Lower and Upper Confidence interval

S13 – Statistical tests of mean FC of TCEMs

Supplementary Table S13a – Intra class correlation of FC on patient level

TCEM_I	
RelPos	ICC (%)
-7	23,6
-6	21,4
-5	19,2
-4	14,2
-3	11,0
-2	14,9
-1	20,9
0	12,0
1	12,0
2	14,4
3	16,2
4	11,7
5	16,0
6	20,2
7	10,8
8	10,8
9	9,9
10	10,9
11	8,4
12	10,7
13	8,3
14	12,4
15	10,5
16	14,1
17	12,7
18	11,3
19	16,6
20	17,9
21	6,9
22	29,1
23	26,3
24	30,4
25	37,8
26	40,0
27	29,2
28	26,3
29	0,0

TCEM_IIa	
RelPos	ICC (%)
-7	15,8
-6	22,5
-5	21,8
-4	13,8
-3	10,3
-2	11,4
-1	8,2
0	21,6
1	9,1
2	11,5
3	13,9
4	14,7
5	18,0
6	9,7
7	10,5
8	10,7
9	13,2
10	14,0
11	8,1
12	7,2
13	9,6
14	8,0
15	11,2
16	12,8
17	13,4
18	10,1
19	9,0
20	32,7
21	16,5
22	21,0
23	24,3
24	25,3
25	29,2
26	25,0
27	0,0
28	31,6
29	57,8
30	0,0

TCEM_IIb	
RelPos	ICC (%)
-7	13,6
-6	19,9
-5	15,5
-4	14,6
-3	24,2
-2	13,6
-1	12,4
0	15,0
1	6,4
2	11,4
3	18,4
4	17,5
5	16,9
6	14,1
7	18,8
8	15,0
9	11,0
10	10,2
11	12,1
12	7,0
13	9,5
14	10,5
15	12,3
16	13,6
17	12,9
18	8,2
19	14,1
20	28,4
21	19,0
22	13,9
23	28,8
24	29,2
25	32,5
26	27,6
27	10,3
28	36,5
29	55,0
30	0,0

ICC=Intra class correlation

Supplementary Table S13b – Mean difference in FC of TCEMI motifs between MS and OIND patients

RelPos	Mean diff	p-value	LCI	UCI	SIGN adj	SIGN
-7	0,636	0,163	-0,257	1,529		
-6	0,381	0,411	-0,528	1,290		
-5	0,031	0,943	-0,809	0,871		
-4	0,006	0,984	-0,596	0,608		
-3	-0,397	0,038	-0,772	-0,022	*	*
-2	-0,441	0,063	-0,906	0,025	*	
-1	-0,342	0,318	-1,012	0,328		
0	-0,019	0,928	-0,425	0,388		
1	0,336	0,134	-0,103	0,775		
2	0,690	0,013	0,144	1,237	*	*
3	0,874	0,008	0,233	1,516	*	*
4	0,674	0,003	0,233	1,116	*	*
5	0,697	0,039	0,036	1,358	*	*
6	0,896	0,032	0,077	1,715	*	*
7	0,447	0,016	0,085	0,808	*	*
8	0,440	0,008	0,115	0,765	*	*
9	0,254	0,101	-0,050	0,557		
10	0,277	0,074	-0,027	0,582	*	
11	0,239	0,033	0,019	0,459	*	*
12	0,322	0,014	0,064	0,580	*	*
13	0,212	0,042	0,008	0,417	*	*
14	0,339	0,009	0,084	0,595	*	*
15	0,165	0,178	-0,075	0,406		
16	0,166	0,322	-0,163	0,495		
17	0,084	0,571	-0,207	0,376		
18	0,070	0,623	-0,208	0,348		
19	0,075	0,702	-0,308	0,457		
20	0,154	0,413	-0,215	0,523		
21	0,094	0,522	-0,195	0,384		
22	0,809	0,028	0,087	1,530	*	*
23	0,406	0,269	-0,314	1,126		
24	0,235	0,510	-0,464	0,935		
25	-0,583	0,261	-1,600	0,433		
26	-0,148	0,847	-1,654	1,358		
27	-0,016	0,987	-1,994	1,962		

LCI=Lower confidence interval. UCI=upper confidence interval. ICC=Intra class correlation
Black stars indicate significant differences. Red stars indicate significant differences after correction for multiple testing (20% FDR, Benjamini-Hochberg)

Supplementary Table S13c – Mean difference in FC of TCEMIIa motifs between MS and OIND patients

RelPos	Mean diff	p-value	LCI	UCI	SIGN adj	SIGN
-7	-0,118	0,717	-0,755	0,520		
-6	0,258	0,568	-0,629	1,145		
-5	0,067	0,873	-0,757	0,892		
-4	-0,229	0,287	-0,651	0,192		
-3	-0,031	0,853	-0,360	0,298		
-2	-0,034	0,854	-0,394	0,326		
-1	0,241	0,052	-0,002	0,485		
0	0,611	0,146	-0,213	1,434		
1	0,348	0,034	0,027	0,670	*	*
2	0,462	0,030	0,046	0,879	*	*
3	0,141	0,640	-0,449	0,730		
4	-0,099	0,762	-0,741	0,543		
5	0,593	0,000	0,533	0,654	*	*
6	0,260	0,177	-0,117	0,636		
7	0,044	0,839	-0,385	0,473		
8	0,437	0,023	0,059	0,815	*	*
9	0,104	0,697	-0,419	0,627		
10	0,394	0,125	-0,109	0,896		
11	0,340	0,005	0,100	0,580	*	*
12	0,041	0,756	-0,217	0,299		
13	0,474	0,000	0,239	0,708	*	*
14	0,219	0,080	-0,026	0,465		
15	0,211	0,202	-0,113	0,534		
16	0,375	0,036	0,025	0,726	*	*
17	-0,045	0,838	-0,480	0,390		
18	0,099	0,569	-0,241	0,439		
19	-0,097	0,576	-0,436	0,243		
20	-0,205	0,743	-1,433	1,022		
21	0,271	0,379	-0,333	0,875		
22	0,641	0,064	-0,036	1,318		
23	-0,024	0,962	-1,016	0,968		
24	0,354	0,470	-0,606	1,315		
25	-0,207	0,760	-1,535	1,121		
26	0,708	0,317	-0,679	2,095		
27	-1,000	0,220	-2,598	0,598		

LCI=Lower confidence interval. UCI=upper confidence interval. ICC=Intra class correlation
Black stars indicate significant differences. Red stars indicate significant differences after correction for multiple testing (20% FDR, Benjamini-Hochberg)

Supplementary Table S13d – Mean difference in FC of TCEMIIb motifs between MS and OIND patients

RelPos	Mean diff	p-value	LCI	UCI	SIGN adj	SIGN
-7	-0,041	0,865	-0,515	0,432		
-6	0,247	0,517	-0,500	0,994		
-5	-0,261	0,369	-0,831	0,308		
-4	-0,173	0,432	-0,605	0,259		
-3	0,394	0,385	-0,495	1,283		
-2	0,167	0,443	-0,260	0,595		
-1	0,288	0,131	-0,086	0,663		
0	0,383	0,152	-0,140	0,906		
1	0,140	0,215	-0,081	0,361		
2	0,371	0,068	-0,027	0,770		
3	-0,066	0,867	-0,843	0,710		
4	-0,139	0,720	-0,897	0,619		
5	0,586	0,092	-0,096	1,268		
6	0,414	0,134	-0,128	0,957		
7	0,359	0,373	-0,431	1,150		
8	0,703	0,006	0,201	1,206	*	*
9	0,303	0,134	-0,094	0,701		
10	0,263	0,145	-0,090	0,616		
11	0,244	0,242	-0,165	0,653		
12	0,059	0,628	-0,180	0,298		
13	0,439	0,000	0,208	0,671	*	*
14	0,089	0,600	-0,244	0,422		
15	0,136	0,480	-0,242	0,515		
16	0,303	0,152	-0,111	0,718		
17	-0,130	0,953	-0,439	0,413		
18	-0,024	0,879	-0,334	0,286		
19	0,085	0,757	-0,451	0,620		
20	-0,105	0,848	-1,177	0,967		
21	0,129	0,722	-0,581	0,839		
22	0,590	0,041	0,025	1,154		*
23	-0,188	0,775	-1,478	1,101		
24	-0,330	0,654	-1,770	1,110		
25	-0,498	0,585	-2,284	1,287		
26	0,811	0,336	-0,843	2,465		
27	-1,635	0,041	-3,203	-0,067		*

LCI=Lower confidence interval. UCI=upper confidence interval. ICC=Intra class correlation
Black stars indicate significant differences. Red stars indicate significant differences after correction for multiple testing (20% FDR, Benjamini-Hochberg)

Supplementary Table S13e – Mean difference in FC of TCEMI motifs between IGHV4 and other IGHV family fragments

RelPos	Mean diff	p-value	LCI	UCI	SIGN adj	SIGN
-7	-0,954	0,000	-1,010	-0,897	*	*
-6	-1,188	0,000	-1,250	-1,125	*	*
-5	-0,890	0,000	-0,955	-0,824	*	*
-4	-0,679	0,000	-0,746	-0,613	*	*
-3	-0,709	0,000	-0,771	-0,647	*	*
-2	-0,567	0,000	-0,621	-0,513	*	*
-1	-0,414	0,000	-0,463	-0,366	*	*
0	-0,040	0,153	-0,094	0,015		
1	0,152	0,000	0,090	0,214	*	*
2	0,156	0,000	0,086	0,225	*	*
3	0,306	0,000	0,233	0,379	*	*
4	0,188	0,000	0,113	0,262	*	*
5	0,401	0,000	0,329	0,473	*	*
6	0,483	0,000	0,414	0,551	*	*
7	0,232	0,000	0,168	0,295	*	*
8	0,273	0,000	0,213	0,334	*	*
9	0,188	0,000	0,129	0,246	*	*
10	0,292	0,000	0,236	0,349	*	*
11	0,309	0,000	0,251	0,367	*	*
12	0,217	0,000	0,157	0,277	*	*
13	0,037	0,250	-0,026	0,099		
14	0,042	0,178	-0,019	0,103		
15	-0,002	0,942	-0,069	0,064		
16	-0,067	0,090	-0,144	0,010	*	
17	0,200	0,000	0,110	0,291	*	*
18	-0,092	0,118	-0,207	0,023	*	
19	0,071	0,284	-0,059	0,202		
20	0,037	0,652	-0,123	0,197		
21	-0,039	0,770	-0,298	0,221		
22	0,011	0,955	-0,373	0,395		
23	-0,166	0,516	-0,667	0,335		
24	0,036	0,937	-0,862	0,935		
25	0,318	0,530	-0,675	1,312		
26	0,184	0,815	-1,353	1,721		
27	-0,112	0,958	-4,307	4,082		

LCI=Lower confidence interval. UCI=upper confidence interval. ICC=Intra class correlation
Black stars indicate significant differences. Red stars indicate significant differences after correction for multiple testing (20% FDR, Benjamini-Hochberg)

Supplementary Table S13f – Mean difference in FC of TCEMIIa motifs between IGHV4 and other IGHV family fragments

RelPos	Mean diff	p-value	LCI	UCI	SIGN adj	SIGN
-7	-0,890	0,000	-0,953	-0,827	*	*
-6	-0,070	0,017	-0,127	-0,013	*	*
-5	-0,530	0,000	-0,585	-0,475	*	*
-4	-0,192	0,000	-0,241	-0,142	*	*
-3	-0,654	0,000	-0,705	-0,603	*	*
-2	-0,301	0,000	-0,352	-0,250	*	*
-1	0,187	0,000	0,135	0,239	*	*
0	0,140	0,000	0,081	0,199	*	*
1	0,187	0,000	0,123	0,251	*	*
2	0,117	0,000	0,051	0,183	*	*
3	0,022	0,533	-0,046	0,089		
4	0,110	0,002	0,042	0,179	*	*
5	0,308	0,000	0,239	0,377	*	*
6	0,299	0,000	0,232	0,366	*	*
7	0,142	0,000	0,075	0,210	*	*
8	0,102	0,003	0,033	0,170	*	*
9	0,132	0,000	0,063	0,201	*	*
10	0,311	0,000	0,242	0,381	*	*
11	0,029	0,414	-0,041	0,100		
12	-0,093	0,014	-0,167	-0,019	*	*
13	0,059	0,142	-0,020	0,137	*	
14	0,155	0,000	0,073	0,237	*	*
15	0,023	0,611	-0,065	0,110		
16	0,084	0,099	-0,016	0,185	*	
17	-0,136	0,036	-0,264	-0,009	*	*
18	0,071	0,372	-0,085	0,227		
19	0,632	0,000	0,443	0,820	*	*
20	-0,158	0,197	-0,398	0,082		
21	-0,123	0,484	-0,467	0,221		
22	0,491	0,050	-0,001	0,982	*	
23	-0,784	0,029	-1,487	-0,082	*	*
24	-0,142	0,850	-1,614	1,329		
25	0,347	0,674	-1,270	1,963		
26	-0,465	0,694	-2,779	1,849		
27	-0,839	0,738	-5,744	4,067		

LCI=Lower confidence interval. UCI=upper confidence interval. ICC=Intra class correlation
Black stars indicate significant differences. Red stars indicate significant differences after correction for multiple testing (20% FDR, Benjamini-Hochberg)

Supplementary Table S13g – Mean difference in FC of TCEMIIb motifs between IGHV4 and other IGHV family fragments

RelPos	Mean diff	p-value	LCI	UCI	SIGN adj	SIGN
-7	-0,486	0,000	-0,541	-0,431	*	*
-6	-0,209	0,000	-0,266	-0,152	*	*
-5	-1,436	0,000	-1,493	-1,378	*	*
-4	-0,107	0,000	-0,155	-0,060	*	*
-3	-0,450	0,000	-0,503	-0,397	*	*
-2	0,162	0,000	0,112	0,212	*	*
-1	0,075	0,004	0,024	0,126	*	*
0	-0,378	0,000	-0,436	-0,321	*	*
1	0,107	0,000	0,049	0,165	*	*
2	-0,054	0,084	-0,116	0,007	*	
3	-0,121	0,000	-0,185	-0,057	*	*
4	0,177	0,000	0,110	0,243	*	*
5	0,199	0,000	0,132	0,266	*	*
6	0,320	0,000	0,255	0,385	*	*
7	0,285	0,000	0,219	0,351	*	*
8	0,299	0,003	0,232	0,366	*	*
9	0,178	0,000	0,112	0,245	*	*
10	0,150	0,000	0,082	0,218	*	*
11	-0,046	0,194	-0,115	0,023		
12	-0,089	0,015	-0,161	-0,018	*	*
13	-0,040	0,306	-0,118	0,037		
14	0,439	0,000	0,358	0,520	*	*
15	-0,069	0,130	-0,158	0,020	*	
16	0,038	0,471	-0,065	0,142		
17	0,140	0,033	0,011	0,268	*	*
18	-0,212	0,010	-0,374	-0,050	*	*
19	0,475	0,000	0,267	0,684	*	*
20	-0,359	0,005	-0,610	-0,108	*	*
21	-0,734	0,000	-1,093	-0,376	*	*
22	0,472	0,101	-0,092	1,036	*	
23	-1,195	0,003	-1,977	-0,414	*	*
24	0,419	0,653	-1,410	2,248		
25	1,108	0,276	-0,885	3,101		
26	-1,142	0,390	-3,748	1,464		
27	-1,344	0,591	-6,249	3,532		

LCI=Lower confidence interval. UCI=upper confidence interval. ICC=Intra class correlation
Black stars indicate significant differences. Red stars indicate significant differences after correction for multiple testing (20% FDR, Benjamini-Hochberg)

S14 – TCEM occurrences in the gut microbiome vs IGHV fragments

Supplementary Figure S14 – TCEM occurrence in the gut microbiome assigned to patients IGHV TCEMs

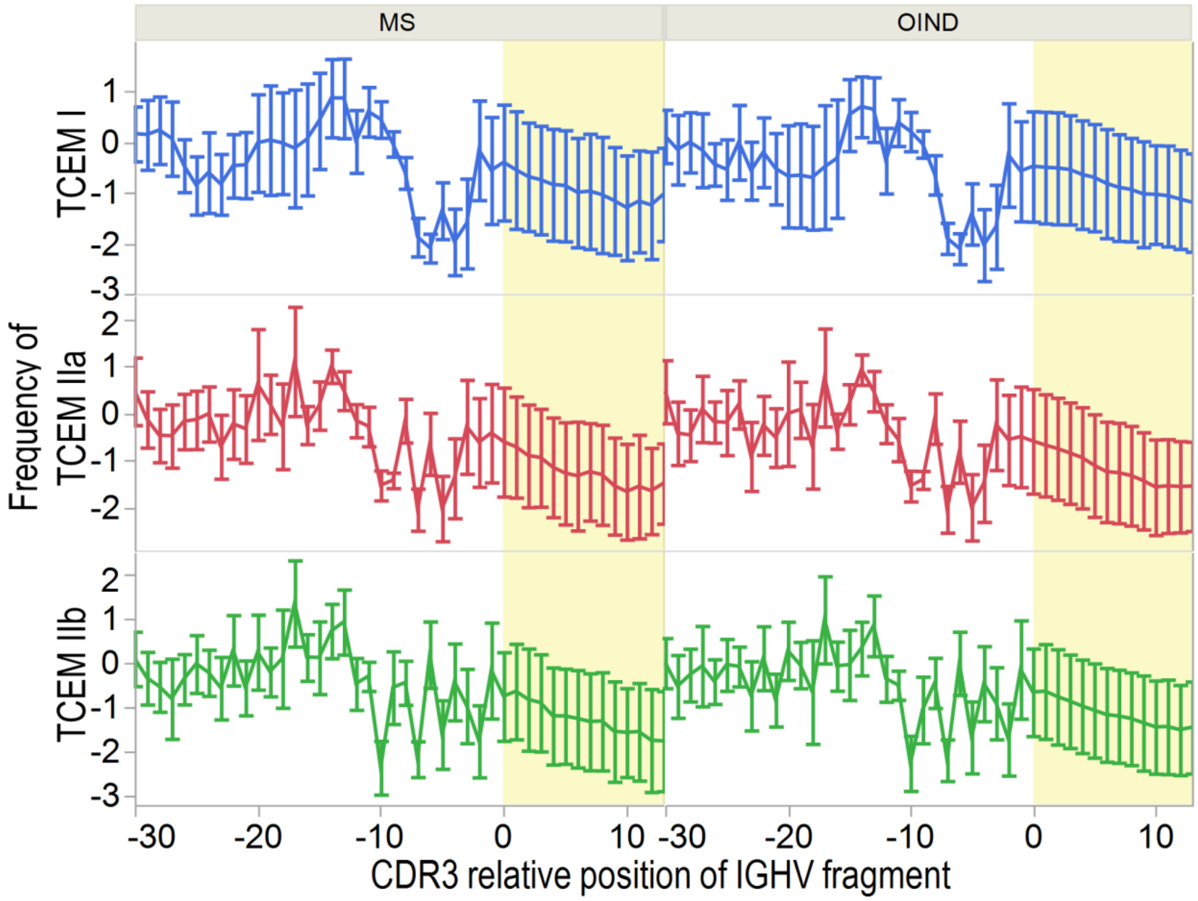

We compared the Johnson standardized occurrence of TCEM in gut microbiome of CDR3- vs FW3-derived fragments by splitting the transcripts at CDR3 relative position -7. Mean Johnson standardized occurrence by IGHV region and by disease are shown as outlier box plots with whiskers covering 1st and 3rd quartile -/+ 1.5*(interquartile range). Supplementary Table S14 shows the adjusted means used for statistical testing. Figure S14 shows the mean Johnson standardized occurrence in gut microbiome in IGHV transcripts by their CDR3 relative positions (first N-terminus amino acid of a 15-mer in the case of TCEM II and a 9-mer in the case of TCEM I). Each error bar is constructed using 1 standard deviation from the mean.

Supplementary Table S14 - Gut microbiome TCEM frequencies in FW3 vs CDR3

		**Johnson SI transformed TCEM I frequency**	**Johnson SI transformed TCEM IIa frequency**	**Johnson SI transformed TCEM IIb frequency**
		ICC (%)	ICC (%)	ICC (%)
**MS**	Patient-level	1.03	1.23	-
	CDR3 relative position	2.84	4.12	3.76
	Transcript clone level	-	-	-
	Mean difference	1.78	0.67	0.53
	LCI	1.76	0.66	0.52
	UCI	1.79	0.68	0.54
	p-value	<0.001	<0.001	<0.001
		Adjusted for cluster effect<br>CDR3 relative position-level	Adjusted for cluster effect<br>CDR3 relative position-level	Adjusted for cluster effect<br>CDR3 relative position-level
		ICC (%)	ICC (%)	ICC (%)
**OIND**	Patient-level	0.34	-	-
	CDR3 relative position	-	3.53	-
	Transcript clone-level	-	-	-
	Mean difference	0.84	0.81	0.72
	LCI	0.84	0.80	0.72
	UCI	0.85	0.81	0.72
	p-value	<0.001	<0.001	<0.001
		Adjusted for cluster effect<br>Patient-level	Unadjusted for cluster effect	Unadjusted for cluster effect

ICC – Intra class correlation, L/UCI – Lower and Upper Confidence interval

S15 – TCEM occurrences in the human proteome vs IGHV fragments

Supplementary Figure S15 – Mean Johnson standardized TCEM counts in the human proteome assigned to patients IGHV fragments TCEMs

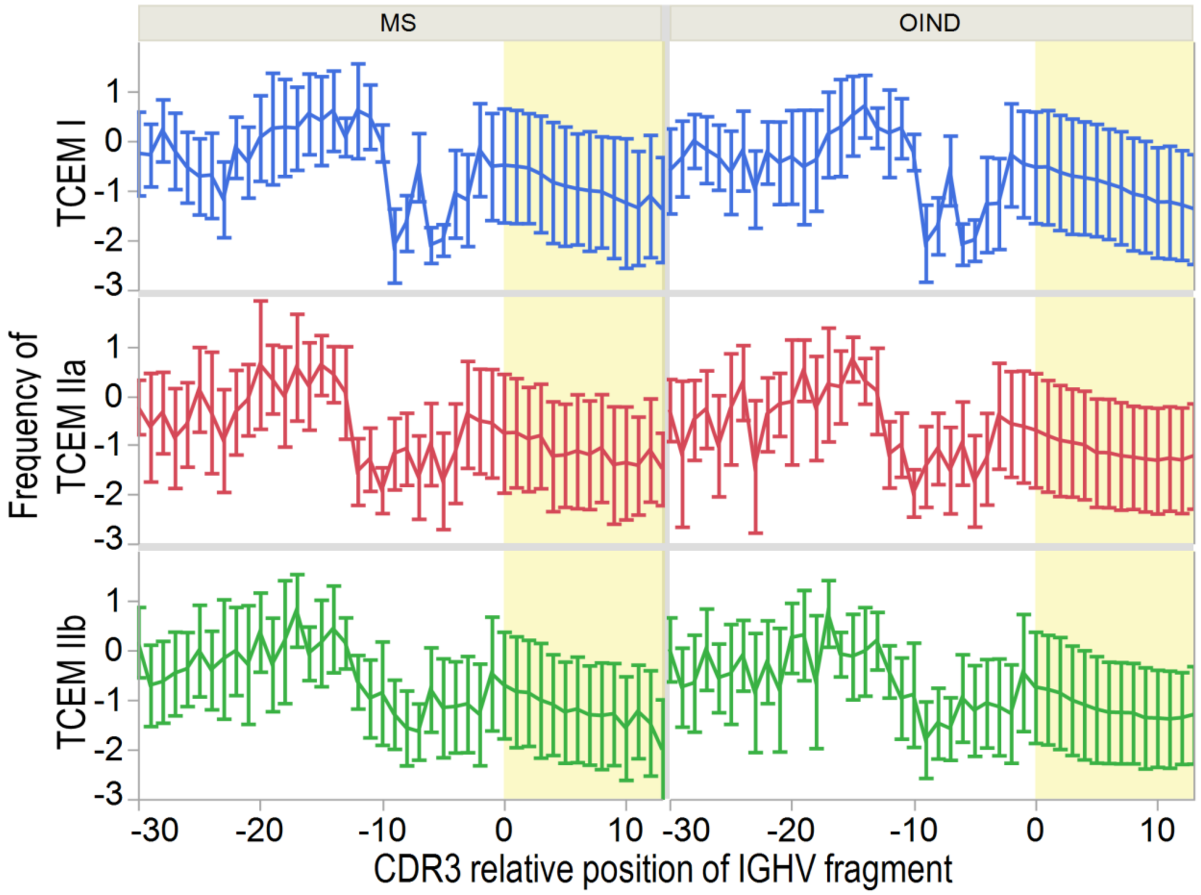

We compared the Johnson standardized occurrence of TCEM in human proteome of CDR3- vs FW3-derived fragments by splitting the transcripts at CDR3 relative position -7. Mean Johnson standardized occurrence by IGHV region and by disease are shown as outlier box plots with whiskers covering 1st and 3rd quartile -/+ 1.5*(interquartile range). Supplementary Table S15 shows the adjusted means used for statistical testing. Figure S15 shows the mean Johnson standardized occurrence in human proteome in IGHV transcripts by their CDR3 relative positions (first N-terminus amino acid of a 15-mer in the case of TCEM II and a 9-mer in the case of TCEM I). Each error bar is constructed using 1 standard deviation from the mean

Supplementary Table S15 - Human proteome TCEM frequencies in FW3 vs CDR3

		**Johnson SI transformed TCEM I frequency**	**Johnson SI transformed TCEM IIa frequency**	**Johnson SI transformed TCEM IIb frequency**
		ICC (%)	ICC (%)	ICC (%)
**MS**	Patient-level	1.49	-	-
	CDR3 relative position	2.71	3.17	3.86
	transcript clone-level	-	-	-
	Mean difference	0.76	0.14	0.25
	LCI	0.75	0.13	0.24
	UCI	0.76	0.15	0.26
	p-value	<0.001	<0.001	<0.001
		Adjusted for cluster effect CDR3 relative position	Adjusted for cluster effect CDR3 relative position	Adjusted for cluster effect CDR3 relative position
		ICC (%)	ICC (%)	ICC (%)
**OIND**	Patient-level	0.34	-	0.35
	CDR3 relative position	-	2.69	3.2
	transcript clone-level	-	-	-
	Mean difference	0.66	0.29	0.20
	LCI	0.65	0.28	0.19
	UCI	0.66	0.30	0.20
	p-value	<0.001	<0.001	<0.001
		Unadjusted for cluster effect	Adjusted for cluster effect CDR3 relative position	Adjusted for cluster effect CDR3 relative position

ICC – Intra class correlation, L/UCI – Lower and Upper Confidence interval
